# Outcomes for implementation science: an enhanced systematic review of instruments using evidence-based rating criteria

**DOI:** 10.1186/s13012-015-0342-x

**Published:** 2015-11-04

**Authors:** Cara C. Lewis, Sarah Fischer, Bryan J. Weiner, Cameo Stanick, Mimi Kim, Ruben G. Martinez

**Affiliations:** Department of Psychological and Brain Sciences, Indiana University, 1101 E. 10th St., Bloomington, IN 47405 USA; Department of Psychiatry and Behavioral Sciences, University of Washington, School of Medicine, Harborview Medical Center, Box 359911, 325 9th Ave, Seattle, WA 98104 USA; 1102-C McGavran-Greenberg Hall, University of North Carolina at Chapel Hill, 135 Dauer Drive, Campus Box 7411, Chapel Hill, NC 27599-7411 USA; Department of Psychology, University of Montana, 32 Campus Dr., Skaggs Bldg. 202, Missoula, MT 59812 USA; North Carolina Translational and Clinical Sciences Institute, University of North Carolina at Chapel Hill, 160 N. Medical Drive, Brinkhous-Bullitt, 2nd Floor, CB# 7064, Chapel Hill, NC 27599-7064 USA; Center for Biobehavioral Health Disparities Research, Duke University, Box 90420, Durham, NC 27708-0420 USA; Department of Psychology, Virginia Commonwealth University, 806 West Franklin St., Richmond, VA 23220 USA

**Keywords:** Systematic review, Implementation, Dissemination, Instruments, Evidence-based assessment, Psychometrics

## Abstract

**Background:**

High-quality measurement is critical to advancing knowledge in any field. New fields, such as implementation science, are often beset with measurement gaps and poor quality instruments, a weakness that can be more easily addressed in light of systematic review findings. Although several reviews of quantitative instruments used in implementation science have been published, no studies have focused on instruments that measure implementation outcomes. Proctor and colleagues established a core set of implementation outcomes including: *acceptability*, *adoption*, *appropriateness*, *cost*, *feasibility*, *fidelity*, *penetration*, *sustainability* (*Adm Policy Ment Health Ment Health Serv Res* 36:24–34, 2009). The Society for Implementation Research Collaboration (SIRC) Instrument Review Project employed an enhanced systematic review methodology (*Implement Sci* 2: 2015) to identify quantitative instruments of implementation outcomes relevant to mental or behavioral health settings.

**Methods:**

Full details of the enhanced systematic review methodology are available (*Implement Sci* 2: 2015). To increase the feasibility of the review, and consistent with the scope of SIRC, only instruments that were applicable to mental or behavioral health were included. The review, synthesis, and evaluation included the following: (1) a search protocol for the literature review of constructs; (2) the literature review of instruments using Web of Science and PsycINFO; and (3) data extraction and instrument quality ratings to inform knowledge synthesis. Our evidence-based assessment rating criteria quantified fundamental psychometric properties as well as a crude measure of usability. Two independent raters applied the evidence-based assessment rating criteria to each instrument to generate a quality profile.

**Results:**

We identified 104 instruments across eight constructs, with nearly half (*n =* 50) assessing *acceptability* and 19 identified for *adoption*, with all other implementation outcomes revealing fewer than 10 instruments. Only one instrument demonstrated at least minimal evidence for psychometric strength on all six of the evidence-based assessment criteria. The majority of instruments had no information regarding responsiveness or predictive validity.

**Conclusions:**

Implementation outcomes instrumentation is underdeveloped with respect to both the sheer number of available instruments and the psychometric quality of existing instruments. Until psychometric strength is established, the field will struggle to identify which implementation strategies work best, for which organizations, and under what conditions.

**Electronic supplementary material:**

The online version of this article (doi:10.1186/s13012-015-0342-x) contains supplementary material, which is available to authorized users.

## Background

Many new scientific fields, like implementation science, are beset with instrumentation issues such as inclusion of oversupply of single-use or adapted instruments that are incommensurable; reliance on instruments with uncertain reliability and validity; and scarcity of instruments for theoretically important constructs [1]. Systematic instrument reviews can help emerging fields address instrumentation issues by utilizing evidence-based, psychometric standards to identify promising instruments needing further testing and areas of needed development for key constructs. While several recent reviews in implementation science have been published [2–4], none have focused on implementation outcomes instruments. This is a significant limitation because implementation outcomes are perhaps the most critical factor in implementation science as they define what we seek to explain in research and what we seek to improve in practice.

Proctor and colleagues [5] articulated the following core set of seven implementation outcomes: *acceptability*, *feasibility*, *uptake*, *penetration*, *cost*, *fidelity*, *and sustainability*. Their conceptual model was updated in 2011 to include *appropriateness* and rename *uptake* as *adoption* [6]. The identification and concrete operationalization of implementation outcomes, separate from service and client outcomes, has clearly shaped the field, earning a total of 479 citations in 5 years and potentially spurring a spike in associated instrument development with 34.1 % of extant implementation outcome instruments developed since 2009 [7]. Yet, the quality of existing implementation outcome instruments remains unclear and perhaps one of the most critical gaps in the literature. Establishing the psychometric properties of implementation outcome instruments is a necessary step to ensuring that predictors, moderators, and mediators of implementation are identified and that comparative effectiveness of implementation strategies is measureable [8].

The primary objective of this review is to assess the reliability, validity, and usability of 104 instruments of implementation outcomes for mental health identified through an enhanced systematic review of the literature performed by and consistent with the goals of the Society for Implementation Research Collaboration (SIRC) Instrument Review Project (IRP) team [7]. Consistent with the mission of SIRC, the review of instruments focused on those directly applicable to mental healthcare and behavioral healthcare settings. In this study, we used a modified version of the evidence-based assessment (EBA) rating criteria developed by Hunsley and Mash [9]. The primary modifications to the established EBA included increasing the number of anchors (from 3 to 5) to promote variability of the ratings and excluding some criteria that are not broadly applicable to the majority of instrument types (e.g., test-retest). The EBA rating criteria were also informed by the work of Terwee and colleagues [10]. The final version of the EBA criteria also incorporated feedback from implementation scientists identified through SIRC; more details on the EBA development process can be found in the project’s methodology paper [7]. Using this revised version of the EBA rating criteria, we assessed the psychometric properties of both published and unpublished instruments for the eight implementation outcomes outlined above [6]. Results highlight instruments that merit consideration for widespread use in addition to areas for recommended development and testing.

## Methods

**Fig. 1:**
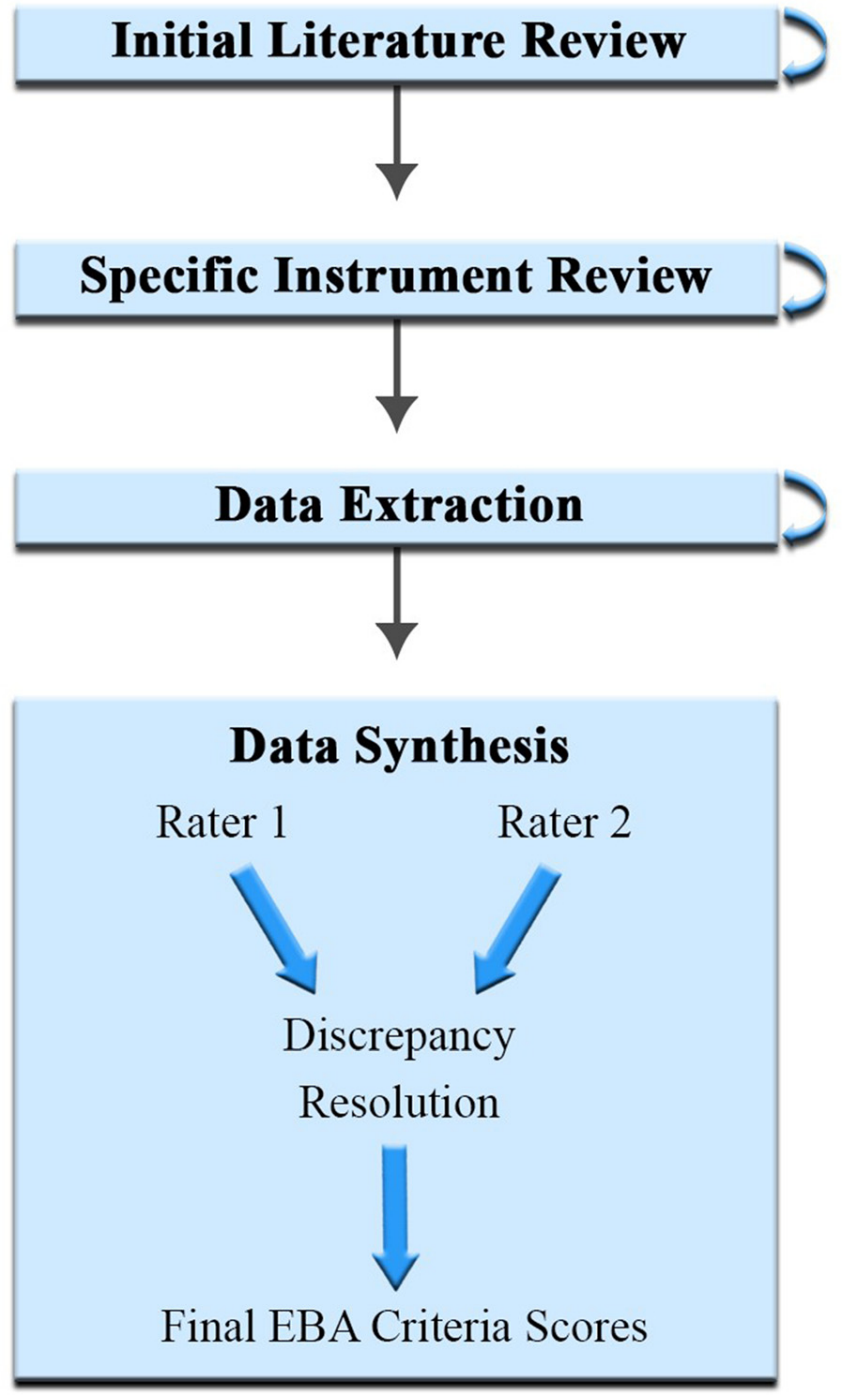
Rating process methodology

The enhanced systematic review methodology for the SIRC Instrument Review Project is described in detail elsewhere [7] and a summary is depicted in Fig. 1. Described below is the methodology as applied to the assessment of constructs in the Implementation Outcomes Framework (IOF).

### Identification of instruments and related published material

For each implementation outcome construct, we performed systematic literature searches of PsycINFO and ISI Web of Science, two widely used bibliographic databases. Our search strings included the construct name (e.g., *adoption*) and synonymous terms (e.g., *uptake*, *utilization*; taken from Proctor and colleagues [6]), as well as terms for *implementation* (e.g., *dissemination*, *quality improvement*), *innovation* (e.g., *evidence-based treatment*), and *instrument* (e.g., *measure*) (see Additional file 1 for search string examples). We limited our search to articles that were written in English, peer-reviewed, published between 1985 and 2012, and measured implementation outcomes quantitatively.

To keep the project manageable and aligned with the project’s funding agency’s priorities, we added the term “mental health” to our search strings. Importantly, the search for *fidelity* instruments was limited to instruments that included either assessments of implementation interventions or instruments that could be applied to any evidence-based practice. This design choice was made because fidelity is one of the only implementation outcomes that has been subjected to extensive reviews, typically focused on specific evidence-based practices (e.g., fidelity to Cognitive Behavior Therapy) [11]. Moreover, because fidelity instruments are typically developed to evaluate specific interventions, their cross-study relevance is limited and thus not a priority for the goals of the SIRC IRP.

A trained research specialist reviewed the titles and abstracts to exclude duplicates and irrelevant articles. Surviving articles were subjected to full-text review, with special attention paid to the method section. The reference section was also scrutinized for articles that might yield additional instruments. A second trained research specialist replicated the construct-focused search and review to ensure consistency and completeness.

To increase the comprehensiveness and exhaustiveness of the search, we employed a respondent-driven, non-probabilistic sampling approach to identify key informants who could help us identify additional instruments in the peer-reviewed literature, the grey literature, or in the later stages of development and testing. This approach, which leverages the informational power of social networks, can augment traditional search methods in situations in which the searched-for items (i.e., instruments) are not clearly and consistently indexed with standard terms in bibliographic databases. We also searched websites and electronic newsletters for additional instruments via search engines such as Google Scholar.

Subsequently, for each instrument, a trained research specialist searched the bibliographic databases for all related published materials, again performing a title and abstract review to ensure the article’s relevance to the project (see Table 1). Inclusion criteria required that articles provided original data about the instrument. All retained published material pertinent to an instrument was then compiled into an instrument packet (i.e., a single PDF). If no related published materials were found, efforts were made to contact the instrument’s author(s). Additional instruments identified in the instrument-focused search and reviews were subjected to the abovementioned strategies for inclusion in the repository. A second research specialist replicated the instrument-focused search and review process to ensure consistency and completeness.

**Table 1:** Literature search strategies

Strategy	Definition
1) Search instrument by name	Full instrument name entered into each search engine.
2) Search instrument by acronym	Acronym(s) entered into each search engine.
3) Search by source article identification	Source article name/reference entered into each search engine.
4) Search by source article “cited by” feature	Source article entered into Google Scholar and “cited by” feature was used.
5) Search for grey literature	Instrument searched in Google to identify grey literature.

### Abstraction of relevant evidence-based assessment information

To facilitate efficiency and consistency in the evidence-based assessment of instruments’ psychometric and pragmatic properties, a team of trained research specialists electronically highlighted and tagged, with searchable key phrases, information within each packet pertinent to six evidence-based assessment (EBA) rating criteria: reliability, structural validity, predictive validity, norms, responsiveness, and usability. The definitions and details of these EBA rating criteria can be found in Additional file 2. The process by which these EBA rating criteria were developed is described elsewhere [7]. Research specialists were trained in the EBA criteria. They highlighted and tagged pilot packets according to EBA rating criteria that were then checked by a project lead and then received additional support from project leads as needed. When the project leads ascertained that the research specialists were performing effectively, the research specialists were permitted to highlight and tag packets independently. Specialists that were ready to double-check the work of others were first subjected to a test (i.e., a complicated packet with errors of omission and commission) to assess their skill. Upon achieving 90 % accuracy on the test, research specialists were permitted to double-highlight packets and monitor the work of more inexperienced specialists.

### Analysis and presentation

Each packet was then sent to two independent raters who were either implementation science experts or advanced research specialists with the guidance of one of the lead authors. As depicted in the guidelines found in Additional file 2, each criterion would receive a rating of “none” (0), “minimal/emerging” (1), “adequate” (2), “good” (3), or “excellent” (4). Raters used the conservative “worst score counts” methodology [12]. If an instrument exhibited “minimal” level of reliability in one study and a “good” level of reliability in another, the rater assigned a “minimal” rating for this criterion. When the raters differed by one point in their ratings, their ratings were averaged unless a clear mistake or misunderstanding occurred. When the raters differed by more than one point, a third expert rated the instrument and adjudicated the discrepancy with the two raters.

Simple statistics (e.g., frequencies) were calculated to describe the availability of information about the psychometric and pragmatic properties of the instruments identified in our review, both overall and by IOF construct. The same procedures were employed to describe the psychometric and pragmatic quality (i.e., the EBA ratings) of the instruments. A total score for each instrument was calculated by summing the EBA ratings for the instrument. Finally bar charts were created to facilitate head-to-head comparisons of instruments by IOF construct. These bar charts allow for visual determination of overall instrument quality, as indicated by the total length of the bars. Simultaneously, the shading allows for a within-criterion comparison of the strength of each instrument with respect to specific criteria.

## Results

### Instrument search results

**Fig. 2:**
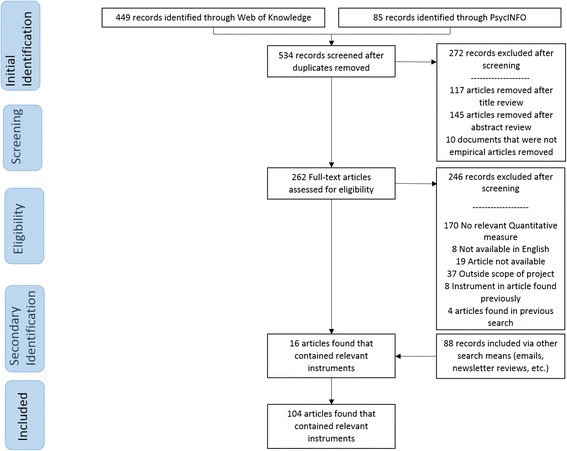
PRISMA enhanced systematic review flowchart for all constructs

**Fig. 3:**
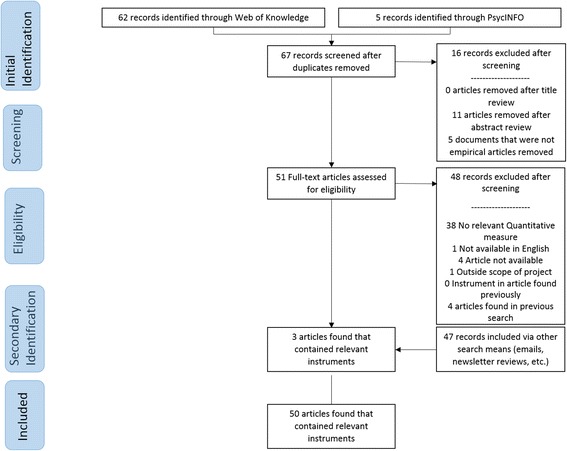
Acceptability PRISMA enhanced systematic review flowchart

**Fig. 4:**
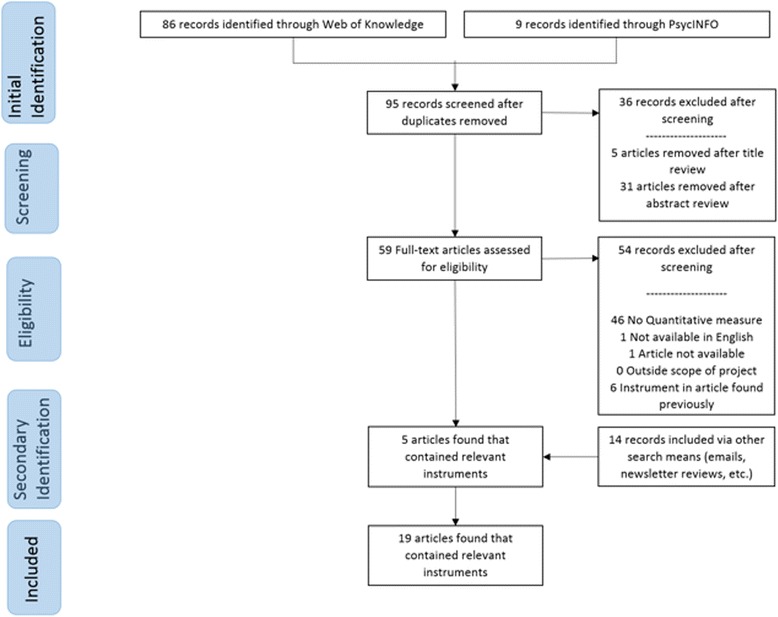
Adoption PRISMA enhanced systematic review flowchart

**Fig. 5:**
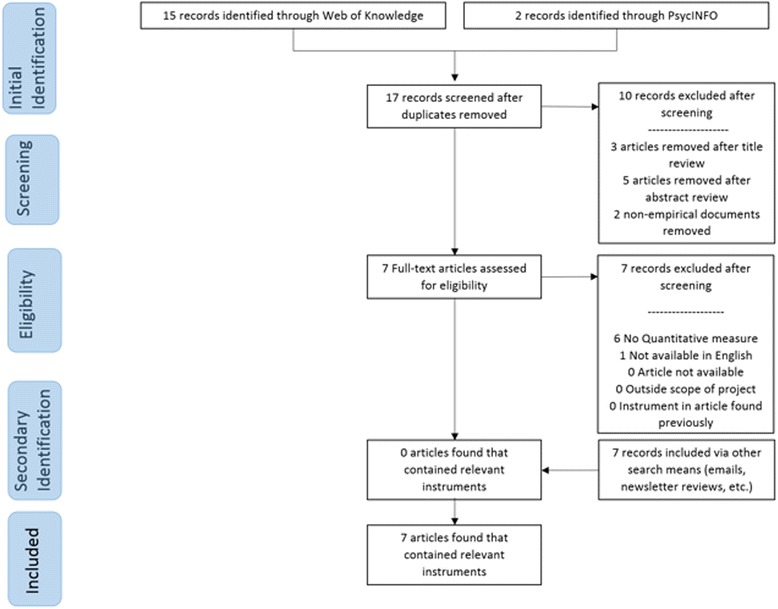
Appropriateness PRISMA enhanced systematic review flowchart

**Fig. 6:**
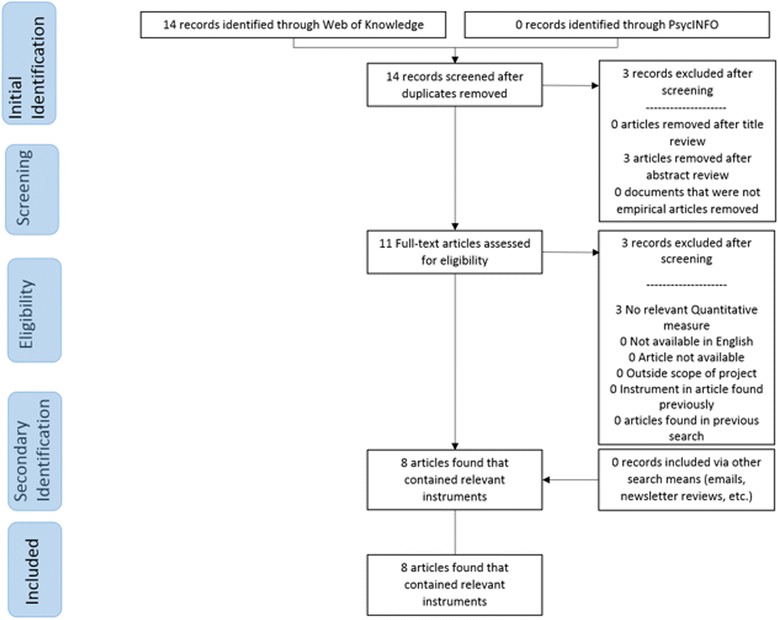
Cost PRISMA enhanced systematic review flowchart

**Fig. 7:**
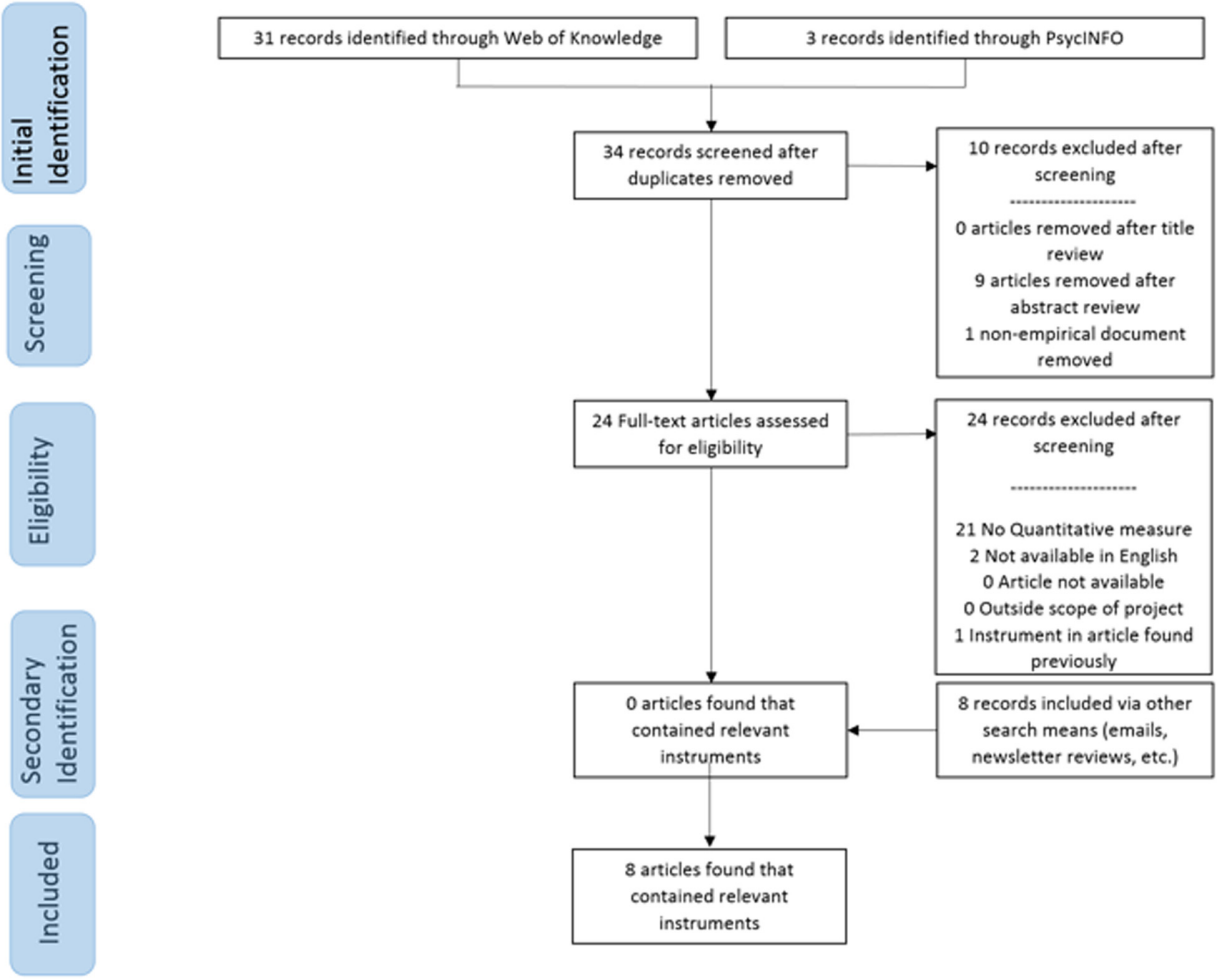
Feasibility PRISMA enhanced systematic review flowchart

**Fig. 8:**
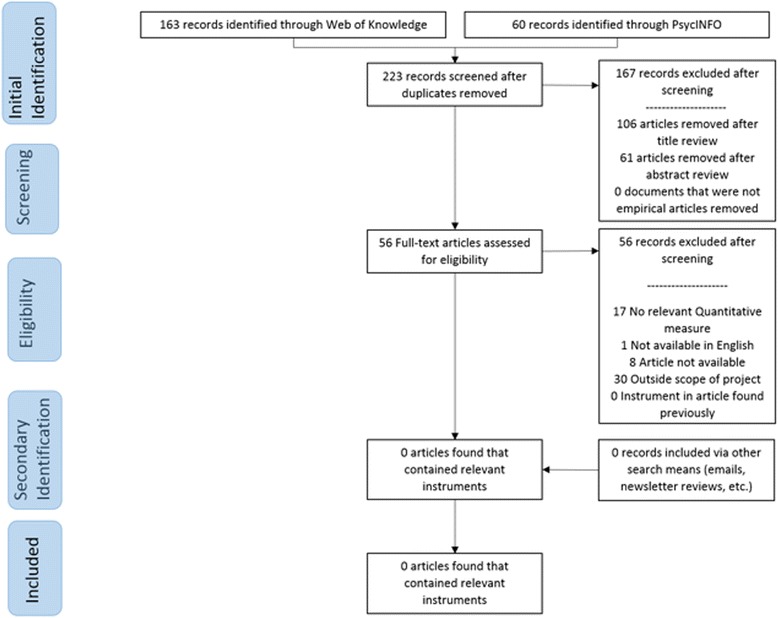
Fidelity PRISMA enhanced systematic review flowchart

**Fig. 9:**
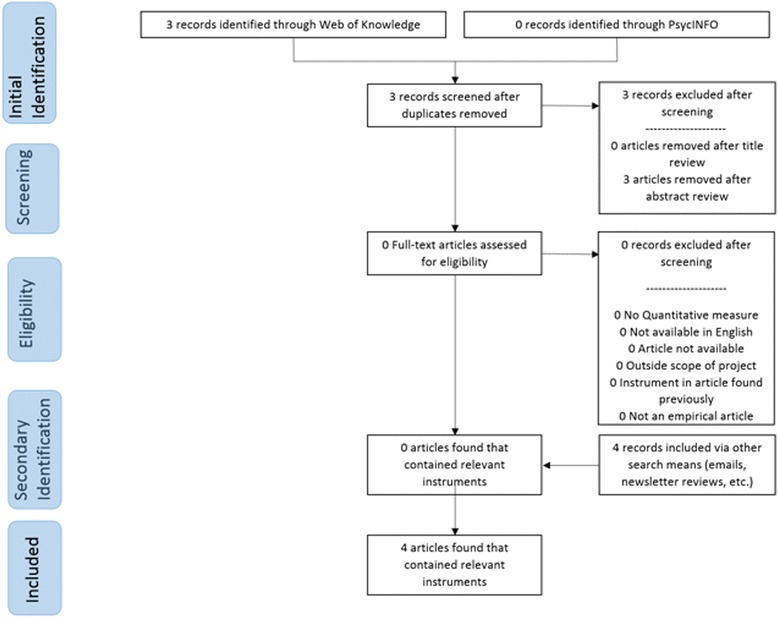
Penetration PRISMA enhanced systematic review flowchart

**Fig. 10:**
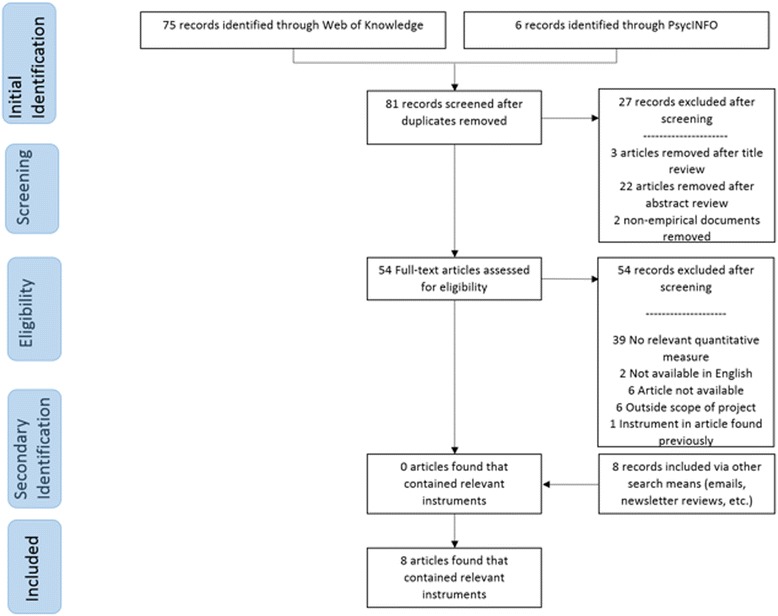
Sustainability PRISMA enhanced systematic review flowchart

Traditional systematic review methods did not prove useful for identifying articles with implementation outcomes instruments. While searches of electronic bibliographic databases yielded 534 unique (non-duplicate) articles (see Fig. 2), only 16 articles were retained following our review process as they had relevant instruments (construct PRISMA flowcharts in Figs. 3, 4, 5, 6, 7, 8, 9, and 10). By contrast, respondent-driven sampling emails and targeted newsletter reviews proved far more useful for instrument identification with a total of 88 unique (non-duplicate) instruments obtained using these methods.

### Evidence-based assessment rating

Overall, availability of information on the psychometric and pragmatic properties across all 104 instruments was limited and variable. Only one instrument identified in our review—the *Levels of Institutionalization Scales for Health Promotion Programs* [13]—had information available for all six EBA rating criteria. Of the remaining 103 instruments, 4 % were missing information for only one rating criteria, 20 % for only two rating criteria, 29 % for three rating criteria, and 46 % for four or more rating criteria. Said differently, less than half of the identified instruments revealed any information pertaining to four of the six EBA criteria. In terms of individual rating criteria, information on reliability was not available for 51 % of instruments, for structural validity 74 %, for predictive validity 82 %, for norms 28 %, and for responsiveness 96 % (Table 2). All instruments had information available for usability, as indicated by a simple item count indicating instrument length.

**Table 2:** Number and percentage of instruments with a rating of 1 or more for each construct

	Rating criteria											
Construct name	Internal consistency		Structural validity		Predictive validity		Norms		Responsiveness		Usability	
	#	%	#	%	#	%	#	%	#	%	#	%
Acceptability	36	72.0	13	26.0	11	22.0	46	92.0	1	2.00	50	100.0
Adoption	8	42.1	7	36.8	5	26.3	10	52.6	0	0.00	19	100.0
Appropriateness	2	28.6	2	28.6	1	14.3	3	42.9	1	14.3	7	100.0
Cost	0	0.00	0	0.00	0	0.00	6	75.0	0	0.00	6	75.0
Feasibility	1	12.5	1	12.5	0	0.00	4	50.0	0	0.00	8	100.0
Penetration	1	25.0	1	25.0	1	25.0	4	100.0	1	25.0	4	100.0
Sustainability	3	37.5	3	37.5	1	12.5	2	25.0	1	12.5	8	100.0

**Fig. 11:**
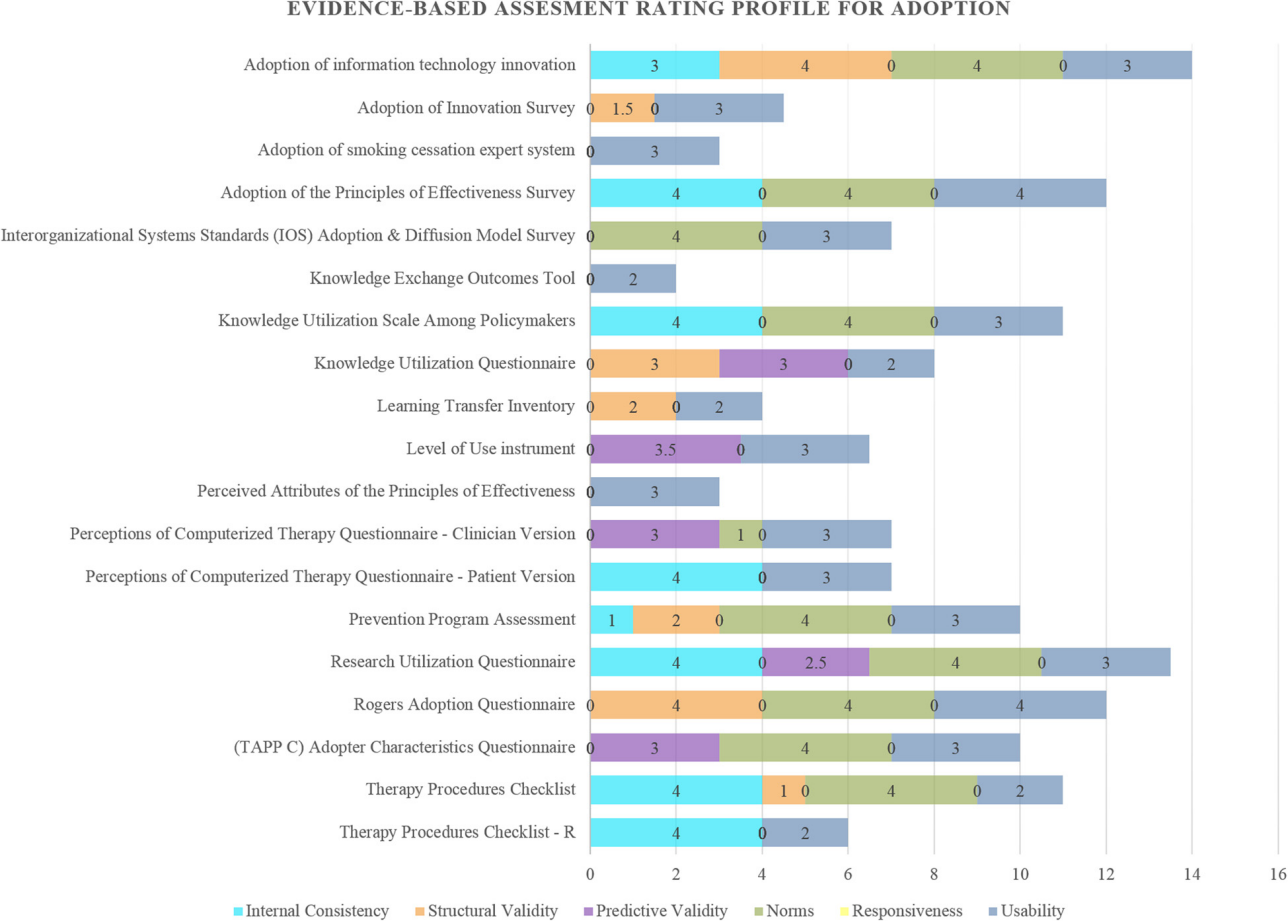
Adoption head-to-head comparison graph

Overall, the psychometric and pragmatic qualities of the instruments identified in our review were modest. The total scores for the six EBA rating criteria ranged from two to 19.5 (Additional file 3); the highest possible total score was 24 with a median total score of eight and a modal total score of seven. In terms of individual rating criteria, the percentage of instruments rated “good” or “excellent” for reliability was 47 %, for structural validity 17 %, for predictive validity 9 %, for norms 53 %, for responsiveness 2 %, and for usability 89 %. Further summary statistics can be found in Tables 2, 3, and 4. Graphs depicting the results by construct can be found in Fig. 11 and Additional file 4: Figures S12–S19. Figure 11 depicts the Evidence-Based Assessment Rating Profile (i.e., head-to-head comparison graph) for the *adoption* construct as an example. All information collected through the review and rating process is available to members of SIRC on our website.^1^

**Table 3:** Summary statistics of all instrument ratings, including scores of “0”

	Rating criteria											
Construct name	Internal consistency		Structural validity		Predictive validity		Norms		Responsiveness		Usability	
	M	SD	M	SD	M	SD	M	SD	M	SD	M	SD
Acceptability	2.66	1.77	0.90	1.57	0.51	1.14	2.88	1.32	0.08	0.57	3.30	0.51
Adoption	1.47	1.90	0.92	1.42	0.79	1.37	1.95	2.01	0.00	0.00	2.84	0.60
Appropriateness	1.00	1.73	0.29	0.49	0.14	0.19	1.29	1.70	0.57	1.51	3.00	0.58
Cost	0.00	0.00	0.00	0.00	0.00	0.00	2.63	1.92	0.00	0.00	2.63	1.77
Feasibility	0.38	1.06	0.50	1.41	0.00	0.00	1.25	1.39	0.00	0.00	3.38	0.52
Penetration	1.00	2.00	1.00	2.00	0.75	1.50	3.25	0.96	0.38	0.75	3.75	0.50
Sustainability	1.25	1.75	0.88	1.46	0.13	0.35	1.00	1.85	0.13	0.35	3.00	0.53

**Table 4:** Summary statistics of all instrument ratings, non-zero ratings only

	Rating criteria											
Construct name	Internal consistency		Structural validity		Predictive validity		Norms		Responsiveness		Usability	
	M	SD	M	SD	M	SD	M	SD	M	SD	M	SD
Acceptability	3.71	0.70	3.46	0.66	2.32	1.31	3.13	1.05	N/A	N/A	3.30	0.51
Adoption	3.50	1.07	2.50	1.90	3.00	0.35	3.70	0.95	N/A	N/A	2.71	0.47
Appropriateness	3.50	0.71	0.50	0.00	N/A	N/A	3.00	1.00	N/A	N/A	3.00	0.58
Cost	N/A	N/A	N/A	N/A	N/A	N/A	3.50	1.22	N/A	N/A	3.50	0.84
Feasibility	N/A	N/A	N/A	N/A	N/A	N/A	2.50	0.58	N/A	N/A	3.38	0.52
Penetration	N/A	N/A	N/A	N/A	N/A	N/A	3.25	0.96	N/A	N/A	3.75	0.50
Sustainability	3.33	0.58	2.33	1.53	N/A	N/A	4.00	0.00	N/A	N/A	3.00	0.53

N/A indicates that the given category had no or only one non-zero score

*Acceptability* (*N* = 50) is the perception among implementation stakeholders that a given treatment, service, practice, or innovation is agreeable, palatable, or satisfactory [6]. Nearly half of the instruments identified in our review measured the *acceptability* of the intervention (*N* = 41) or the implementation process (*N* = 9) (see Additional file 3: Tables S5 and S6). Information on reliability was available for 72 %, structural validity for 26 %, predictive validity for 22 %, norms for 92 %, responsiveness for 2 %, and usability for 100 %. Among those instruments for which psychometric information was available (i.e., those with non-zero ratings), the median rating was “4—excellent” for reliability, “4—excellent” for structural validity, “2—adequate” for predictive validity, “4—excellent” for norms, and “3—good” for usability. Only one instrument received a non-zero score on responsiveness, with a score of “4—excellent”. The Pre-Referral Intervention Team Inventory [14] had the highest overall rating among intervention *acceptability* instruments (total score = 18.5), with “4—excellent” ratings for reliability, structural validity, and norms; and “3—good” ratings for predictive validity and usability. The Practitioner’s Attitudes toward Treatment Manuals [15] had the highest overall rating among implementation process *acceptability* instruments (total score = 17), with “4—excellent” ratings for reliability, structural validity, and norms; “3—good” rating for usability; and “2—adequate” rating for predictive validity.

*Adoption* (*N* = 19) is the intention, initial decision, or action to try or employ an innovation or evidence-based practice [6]. About 20 % of the instruments identified in our review measured *adoption* (see Additional file 3: Table S7). Information on reliability was available for 42 %, structural validity for 36 %, predictive validity for 26 %, norms for 53 %, responsiveness for 0 %, and usability for 100 %. Among those instruments with accessible psychometric information, the median rating was “4—excellent” for reliability, “2—adequate” for structural validity, “3—good” for predictive validity, “4—excellent” for norms, and “3—good” for usability. All *adoption* instruments received a responsiveness score of “0—no evidence”. The Adoption of Information Technology Innovation [16] scale had the highest overall rating (score = 14), with “4—excellent” ratings for structural validity and norms and “3—good” ratings for reliability and usability; however, no information about predictive validity is available. The Research Utilization Questionnaire [17] was also notable for its high overall rating (total score = 13.5).

*Appropriateness* (*N* = 7) is the perceived fit, relevance, or compatibility of the innovation or evidence based practice for a given practice setting, provider, or consumer; and/or perceived fit of the innovation to address a particular issue or problem [6]. Seven percent of the instruments identified in our review measured *appropriateness* (see Additional file 3: Table S8). Information on reliability was available for 29 %, structural validity for 29 %, predictive validity for 14 %, norms for 43 %, responsiveness for 14 %, and usability for 100 %. Among those instruments for which psychometric information was available, the median rating was “3.5—good-excellent” for reliability, “0.5—none-minimal” for structural validity, “3—good” for norms, and “3—good” for usability. Only one instrument provided a non-zero score for predictive validity, with a reported score of “0.5—none-minimal”. One instrument provided a non-zero score for responsiveness, with a score of “4—excellent”. The Parenting Strategies Questionnaire [18] had the highest overall rating (total score = 14), with “4—excellent” ratings for reliability, responsiveness, and usability, and “2—adequate” rating for norms; however, no information is available about structural or predictive validity.

*Cost* (*N* = 8) is the financial impact of an implementation effort [6]. Eight percent of the instruments identified in our review measured *cost* (see Additional file 3: Table S9). *Cost* is not typically treated as a latent construct; consequently, information was not available for reliability or structural validity. Information was also not available for predictive validity or responsiveness on any of the identified cost instruments. Information on norms and usability was available for 75 % of *cost* instruments. The two instruments with the highest overall ratings were The Drug Abuse Treatment Cost Analysis Program [19] (total score = 8) and the Utilization and Cost Questionnaire [20] (total score = 8).

*Feasibility* (*N* = 8) is the extent to which a new treatment, or an innovation, can be successfully used or carried out within a given agency or setting [6]. Eight percent of the instruments identified in our review measured *feasibility* (see Additional file 3: Table S10). Information on reliability was available for 12 %, structural validity for 12 %, predictive validity for 0 %, norms for 50 %, responsiveness for 0 %, and usability for 100 %. Among those instruments for which psychometric information was available, the median rating was “2.5—adequate-good” for norms and “3—good” for usability. All instruments received scores of “0—no evidence” for predictive validity and responsiveness. There was one non-zero score for reliability (score of “3—good”) and one non-zero score for structural validity (score of “4—excellent”). The Measure of Disseminability [21] had the highest overall rating (total score = 10), with “4—excellent” rating for structural validity and “3—good” ratings for reliability and usability; however, no information is available about predictive validity, norms, or responsiveness.

*Fidelity* (*N* = 0) is the degree to which an intervention was implemented as it was prescribed in the original protocol or as it was intended by the program developers [6]. No *fidelity* instruments were identified in our review that included either assessments of implementation interventions (e.g., instruments that measure frequency and structure of an evidence-based practice training) or instruments that could be applied to any evidence-based practice. *Fidelity* instruments for specific clinical interventions were not considered for inclusion in the repository at this time.

*Penetration* (*N* = 4) is the integration of a practice within a service setting and its subsystems [6]. Four percent of the instruments identified in our review measured *penetration*. Information on reliability was available for 25 %, structural validity for 25 %, predictive validity for 25 %, norms for 100 %, responsiveness for 0 %, and usability for 100 %. All instruments but one received scores of “0—no evidence” for internal consistency, structural validity, predictive validity, and responsiveness (see Additional file 3: Table S11), meaning that simple statistic calculations could only be completed for norms and usability. Information on norms and usability was available for all four instruments, with the median rating of “3—good” for norms and “4—excellent” for usability. The Levels of Institutionalization Scale [13] had the highest overall rating (total score = 19.5), with “4—excellent” ratings for reliability, structural validity, and norms; “3—good” ratings for predictive validity and usability; and “1—emerging” to “2—adequate” rating for responsiveness.

*Sustainability* (*N* = 8) is the extent to which a newly implemented treatment is maintained or institutionalized within a service setting’s ongoing, stable operations [6]. Eight percent of the instruments identified in our review measured *sustainability* (see Additional file 3: Table S12). Information on reliability was available for 38 %, structural validity for 38 %, predictive validity for 13 %, norms for 25 %, responsiveness for 13 %, and usability for 100 %. Among those instruments for which psychometric information was available, the median rating was “3—good” for reliability, “2—adequate” for structural validity, “4—excellent” for norms, and “3—good” for usability. One instrument received a non-zero score for predictive validity (score of “1—minimal/emerging”) and one instrument received a non-zero score for responsiveness (score of “1—minimal/emerging”). The School-Wide Universal Behavior Sustainability Index-School Teams scale [22] had the highest overall rating (total score = 16), with “4—excellent” rating for reliability, structural validity, and norms; “3—good” rating for usability; and “1—emerging” rating for predictive validity. No instruments yielded information relevant for responsiveness.

## Discussion

### The state of instrumentation for implementation outcomes

**Fig. 12:**
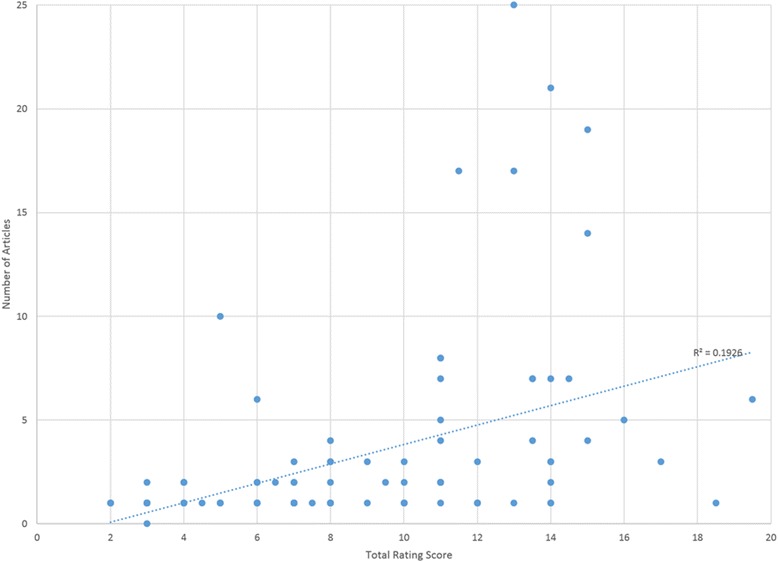
Packet size and total EBA rating score

The findings from this review indicate that there is an uneven distribution of instruments across implementation outcomes for mental and behavioral health. One hypothesis for this is that the number and quality of instruments hinges upon the history and degree of theory and published research available for a particular construct. Indeed, there was a significant positive correlation with the published literature available for a particular outcome and the instrument quality rating (*r =* 0.439, *p* < .001; see Fig. 12). For instance, there is a longstanding focus on treatment *acceptability* in both the theoretical and empirical literature, thus it is unsurprising that *acceptability* (of the intervention) is the most densely populated implementation outcome with respect to instrumentation. However, *sustainability* is a relatively new construct, at least with respect to evidence-based practices, and accordingly few *sustainability* instruments exist.

In addition, some implementation outcomes lend themselves to unique forms of quantitative instruments that fell outside the scope of this review. For instance, *fidelity* instruments tend to be specific to an intervention and thus are not relevant to the field, broadly speaking. That is not to say that *fidelity* instruments that are psychometrically strong do not exist, or that they cannot be applied to multiple studies or contexts. In fact, fidelity instruments may be the most densely populated implementation outcome, perhaps with the highest quality instruments, given the intensity of focus on intervention fidelity in efficacy and effectiveness research, both of which has a much longer history than implementation research. Other outcomes such as *cost* and *penetration* do not reflect latent constructs and so these outcomes are best measured through formula-based instruments that cannot be adequately assessed using our EBA criteria. Finally, there remains conceptual ambiguity and overlap among implementation outcomes (e.g., *acceptability* and *appropriateness*), which has resulted in some instruments including items that arguably measure different constructs.

In order to advance the field and address the issue of uneven distribution of instruments, we recommend increased emphasis on the underdeveloped outcomes where few instruments exist, notably *feasibility*, *appropriateness*, and *sustainability.* Moreover, as noted by Martinez and colleagues [1], careful domain delineation (developing a nomological network) is critical to properly define the construct and limit ambiguity in instrument development.

### The importance, but lack, of psychometric property information

Limited psychometric information was available across the 104 instruments reviewed, with 46 % of identified instruments missing information on four or more of the psychometric properties under investigation. This finding is not unique to implementation outcome instruments; previous reviews of implementation science-related instruments similarly found that 48.4 % [3] lacked criterion validity and 49.2 % [4] lacked evidence of any psychometric validation. It is possible that a reporting problem could explain this finding. For instance, in our study, internal consistency was the most frequently reported psychometric statistic and indeed it is typical for many peer-reviewed journals to require internal consistency reporting. However, it is rarely the case that reporting of other psychometrics is required. Most likely is the case that this finding is a result of few instruments having been subjected to rigorous instrument development and testing processes. There are at least three reasons why this may be the case.

One, instruments are often generated “in-house” and are used just once to suit the specific, immediate needs of a project (of which instrument development is rarely the focus). Two, researchers rely on previously reported psychometric information. Three, researchers do not have the requisite expertise to employ systematic development and testing procedures, such as factor analysis. Consistent with recommendations made by Martinez and colleagues [1], these findings highlight the importance of adopting clear and consistent reporting standards and taking systematic approaches to instrument development, without which the quality of implementation outcomes instrumentation will likely remain poor.

### Relatively low psychometric quality of instruments

Between 98 % (responsiveness) and 47 % (norms) of instruments demonstrated less than “adequate” ratings on the psychometric properties included in the EBA criteria. The properties with the poorest ratings were responsiveness, predictive validity, and structural validity. Low quality ratings on these criteria are likely due to the need for large samples, longitudinal designs, and more sophisticated analytic skills. These challenges may be difficult to overcome. However, the field can work together to establish the psychometric properties of existing instruments. Thus, we recommend that instruments with promising psychometric properties on some criteria be prioritized for further psychometric testing rather than focusing solely on new instrument development.

### Can instruments be both psychometrically strong and pragmatic?

Finally, *usability* is a crude metric for characterizing the “pragmatic” or practical properties of an instrument [23]. In this initial review of IOF instrumentation, we viewed instrument length as relevant to designing and evaluating implementation initiatives outside of the research enterprise given similar literature on provider-reported barriers to utilizing EBA instruments for client outcomes [9, 24]. However, that the majority of instruments consisting of between 11 and 49 items (a rating reflecting “good”) may actually reflect unfeasible instrument length in a practical implementation context. Our team has prioritized developing a stakeholder-driven operationalization of the “pragmatic” construct as it pertains to implementation science instrumentation within the context of an NIMH-funded R01 award. Subsequently, all instruments included in this report will be re-assessed for their pragmatic qualities to determine whether instruments can be both pragmatic and psychometrically strong—a necessary balance to advance both the science and practice of implementation.

### Implications for searching instruments only in traditional databases

Important to highlight is also the disparity in instruments identified via traditional literature databases and the grey literature, with the former producing only 14 % of the instruments identified. The “enhanced” nature of the search process proved critical to uncovering the implementation outcomes instrumentation landscape. Although difficult to confirm the primary reason for this observation, at least two possibilities may explain why traditional database searches yielded so few instruments. First, many of the instruments identified were those that may be best described as “in development” or “single use.” That is, these instruments were not developed via gold standard test development procedures and were not intended to be promoted for use beyond one study. Acknowledging that the instruments were not of high quality was one of the most common reasons instrument authors declined providing their instrument for our website. The general poor quality of the state of implementation outcome instrumentation further substantiates this hypothesis. Second, although some literature databases have a search for “measures” inclusion criteria, our library scientist indicated that the article tagging, according to these parameters, is likely to be invalid given that it is a much more challenging exercise than simply tagging for explicitly listed keywords. Moreover, it is important to note that article reviews that rely on title and abstract are inappropriate for instrument reviews, given that instrument names or references are likely to be embedded in the article text rather than explicitly in the review title or abstract. To avoid developing redundant instruments and to increase the opportunities for the field to collectively establish the psychometrics of newly developed instruments, it is recommended that implementation scientists upload or share their instruments with our evolving SIRC repository or the Grid-Enabled Measures Project [25].

### Limitations

Several limitations are worth noting. First, the methodology employed (described elsewhere; [7]), may be difficult to replicate. That is, the respondent-driven sampling, grey literature, and newsletter reviews generated the majority (85 %) of the instruments found in this study. We found this component of the search method is critical to obtain instruments in development and circumvent the need for consistent keyword tagging of databases. However, there are likely other listservs that may have resulted in access to additional instruments that went overlooked based on our prior knowledge networks. Second, our review did not distinguish between nomothetic instrumentation (i.e., instruments in which interpretation of results are based on comparisons to aggregated data for the instrument) and idiographic instrumentation (i.e., individually selected or tailored instruments of variables or functionality that maximize applicability to individuals or context) [26]. Nomothetic measurement approaches provide the benefit of cross-study comparison. However, the preponderance of single use instruments may reflect a valid argument for the need to employ idiographic methods to optimally investigate implementation efforts. The need for nomothetic versus idiographic instrumentation approaches is an empirical question. Third, our review focused on mental health-relevant implementation instrumentation. This may limit the applicability of the results to implementation scientists or stakeholders from fields outside of mental health or behavioral healthcare. However, implementation science is often described as transdisciplinary in nature such that the outcomes relevant to implementation in mental health and behavioral healthcare are likely to remain applicable regardless of discipline. Fourth, although our EBA rating criteria are intended to be broadly applicable and highlight primary dimensions reflective of the strength of instruments, we did not include a comprehensive array of psychometric properties. For instance, we did not include other forms of reliability such as test-retest and inter-rater reliability, nor did we include other forms of validity such as convergent and divergent validity in our rating criteria. The decision to exclude these properties was made during our pilot testing of the EBA criteria [7] in order to keep the rating process manageable (i.e., brief and focused) and to prioritize the fundamental psychometric properties necessary for quality instrumentation.

### Future directions

Future research should consider (1) increasing the availability of instruments with promising psychometric properties to further establish their quality and (2) populating the underdeveloped constructs with instruments using guidance from a recent publication [1]. Ultimately, this work may elicit focus on the important areas of implementation outcome instrumentation development. Indeed, this enhanced systematic review led to an NIMH-funded R01 in which we seek to advance implementation science through instrument development and evaluation. We will prioritize instrument development of the *acceptability*, *appropriateness*, and *feasibility* constructs given their important relevance in the field as predictors of *adoption* [27]. In addition, we will further innovate the EBA rating criteria by developing the usability criterion into a more complex and comprehensive stakeholder-informed pragmatic criteria drawing upon Glasgow and Riley’s work [23].

## Conclusions

There is a clear need for coordination of instrumentation development focused on implementation outcomes, as highlighted by our results and similar findings from a related project—the Grid Enabled Measures project led by the National Cancer Institute [25]. Although constructs such as *acceptability* appear saturated with instruments, the majority of implementation outcomes are underdeveloped, yielding few instruments or those without evidence of psychometric strength. Without high-quality instruments, it will be difficult to determine predictors, moderators, and mediators of implementation success. Careful attention must be paid to systematic development and testing procedures in addition to the necessary development of articulating instrument reporting standards [1].

## Additional files

Additional file 1:
**Search Strings.**
Additional file 2:
**Evidence Based Assessment Criteria Guidelines.**
Additional file 3:
**Implementation Outcome Rating Scores.** (Tables S5-S12) Additional file 4:
**Construct Head-to-Head Ratings Comparison Graphs.** (Figures S12-S19)

## Abbreviations

CFIR: Consolidated Framework for Implementation Research
DIS: Dissemination and Implementation Science
EBA: evidence-based assessment
GEM: Grid-Enabled Measures Project
IOF: Implementation Outcomes Framework
IRP: Instrument Review Project
NIH: National Institutes of Health
NIMH: National Institute of Mental Health
PRISMA: Preferred Reporting Items for Systematic Reviews and Meta-Analyses
RA: research assistant
SIRC: Formerly known as Seattle Implementation Research Conferences; now Society for Implementation Research Collaboration
SIRC IRP: SIRC Instrument Review Project

## Footnotes

1 Anyone can register to be a SIRC member at societyforimplementationresearchcollaboration.org and thus have access to the repository.
