# Supplementary material for: Outcomes for implementation science: an enhanced systematic review of instruments using evidence-based rating criteria
Source: Implement Sci. 2015 Nov 4;10:155. doi: 10.1186/s13012-015-0342-x (PMC4634818; doi:10.1186/s13012-015-0342-x)
Supplement: Additional file 1: — Search Strings. [file 13012_2015_342_MOESM1_ESM.pdf]

Additional File 1: Search Strings and Strategies

Search String Parameters for Implementation Outcomes Framework Literature Review

---

1. (implement* OR adopt* OR "quality improv*" OR diffus* OR disseminat*)

AND

2. ("empirically supported treatment" OR "evidence based practice" OR "evidence based treatment" OR "evidence based intervention" OR innovation OR guideline)

AND

3. (measure OR instrument OR survey OR questionnaire)

AND

4. (“mental health” OR “behavioral health” OR “behavioural health” OR health)

AND

5. (construct OR synonym)

    e.g., (acceptability OR satisfaction OR agreeable);

    (adoption OR “knowledge translation” OR uptake OR “intention to adopt”);

    (appropriateness OR applicability OR compatibility OR “perceived fit” OR fitness OR sustainability);

    (feasibility OR transferability OR applicability OR practicability OR workability);

    (penetration or “integration of practice” OR infiltration);

    (sustain* OR maintenance OR "long-term implementation" OR routinization OR discontinuation OR "de-adoption" OR durability OR institutionalization OR "capacity building")

Note. “*” tells the search engine to return alternate spellings for a word at the point that the asterisk appears.
