# Supplementary material for: Outcomes for implementation science: an enhanced systematic review of instruments using evidence-based rating criteria
Source: Implement Sci. 2015 Nov 4;10:155. doi: 10.1186/s13012-015-0342-x (PMC4634818; doi:10.1186/s13012-015-0342-x)
Supplement: Additional file 2: — Evidence Based Assessment Criteria Guidelines. [file 13012_2015_342_MOESM2_ESM.pdf]

Additional File 2: Evidence Based Assessment Criteria Guidelines

**Reliability Information**	
0	None (N): α values are not yet available OR are only available for subscales.
1	Minimal/Emerging (M): α values of < .60
2	Adequate (A): α values of .60 - .69
3	Good (G): α values of .70 - .79
4	Excellent (E): α values of ≥ .80
NA	Internal Consistency measures are not applicable for this instrument; OR, classical test theory anchors are not appropriate, results reported using item response theory.
**Structural Validity**	
0	None (N): No exploratory or confirmatory analysis has yet been performed, nor have any Item Response Theory tests of (uni-) dimensionality have been conducted. OR, percent variance explained is not reported.
1	Minimal/Emerging (M): The sample consisted of less than 5 times the number of items AND an exploratory factor analysis explained less than 25% of the variance.
2	Adequate (A): The sample consisted of 5 times the number of items but is less than 100 in total AND<br>an exploratory factor analysis explained less than 50% of the variance OR<br>a confirmatory factor analysis revealed an RMSEA or SRMR of = .08 to .05 OR<br>CFI or GFI = .90 to .95
3	Good (G): The sample consisted of 5 times the number of items and is greater than or equal to 100 in total OR the sample consisted of 5-7 times the number of items but is less than 100 in total AND in either case<br>an exploratory factor analysis explained less than 50% of the variance OR<br>a confirmatory factor analysis revealed an RMSEA or SRMR of = .05 to .03 OR<br>CFI or GFI = .95 to .97
4	Excellent (E): The sample consisted of 7 times the number of items and is greater than 100 in total AND an exploratory factor analysis<br>explained greater than 50% of the variance OR<br>a confirmatory factor analysis revealed an RMSEA or SRMR of < .03 OR<br>GFI or CFI > .97

Criterion (Predictive) Validity Information	
0	None (N): Predictive validity not yet tested or failed to be detected in evaluation.
1	Minimal/Emerging (M): Evidence of small correlation (range: 0.1 to 0.29) between instrument and scores on another test (measuring a distinct construct of interest or outcome) administered at some point in the future.
2	Adequate (A): Evidence of medium correlation (range: 0.3 to 0.49) between instrument and scores on another instrument (measuring a distinct construct of interest or outcome) administered at some point in the future.
3	Good (G): Evidence of strong correlation (range: 0.5 to 1.00) between instrument and scores on another instrument (measuring a distinct construct of interest or outcome) administered at some point in the future.
4	Excellent (E): Evidence of medium-strong correlation (0.3 or higher) between instrument and scores on at least two other instruments (measuring a distinct construct of interest or outcome) administered at some point in the future.
**Norms**	
0	None (N): Norms are not yet available.
1	Minimal/Emerging (M): Measures of central tendency and distribution for the total score (and subscales if relevant) based only on a small (n < 30) sample are available.
2	Adequate (A): Measures of central tendency and distribution for the total score (and subscales if relevant) based on a moderate (n = 30-49) sample are available.
3	Good (G): Measures of central tendency and distribution for the total score (and subscales if relevant) based on a medium (n = 50-99) sample are available.
4	Excellent (E): Measures of central tendency and distribution for the total score (and subscales if relevant) based on a large (n > 100) sample are available.
**Responsiveness (Sensitivity to Change)**	
0	None (N): The instrument has either not been administered both pre- and post-implementation to evaluate sensitivity to change, OR it has been administered and it did not demonstrate responsiveness (change) across an implementation process.
1	Minimal/Emerging (M): The instrument demonstrated change over time based on a small (n < 50) sample.
2	Adequate (A): The instrument demonstrated EITHER clinically OR statistically significant change over time based on a medium sample (n > 50 but < 100).
3	Good (G): The instrument demonstrated change over time reflective of BOTH clinically and statistically significant change based on a large sample (n > 100).
4	Excellent (E): The instrument demonstrated BOTH clinically and statistically significant change over time based on at least two large (n >100) samples.

**Usability (Instrument Length)**	
0	None (N): The instrument is not in the public domain.
1	Minimal (M): The instrument has greater than 100 items.
2	Adequate (A): The instrument has greater than 50 items but fewer than 100.
3	Good (G): The instrument has greater than 10 items but fewer than 50.
4	Excellent (E): The instrument has fewer than 10 items.
