# Supplementary material for: Outcomes for implementation science: an enhanced systematic review of instruments using evidence-based rating criteria
Source: Implement Sci. 2015 Nov 4;10:155. doi: 10.1186/s13012-015-0342-x (PMC4634818; doi:10.1186/s13012-015-0342-x)
Supplement: Additional file 4: — Construct Head-to-Head Ratings Comparison Graphs. (Figures S12-S19) [file 13012_2015_342_MOESM4_ESM.pdf]

Additional File 4: Construct Head-to-Head Ratings Comparison Graphs

Figure 12: Acceptability-Intervention

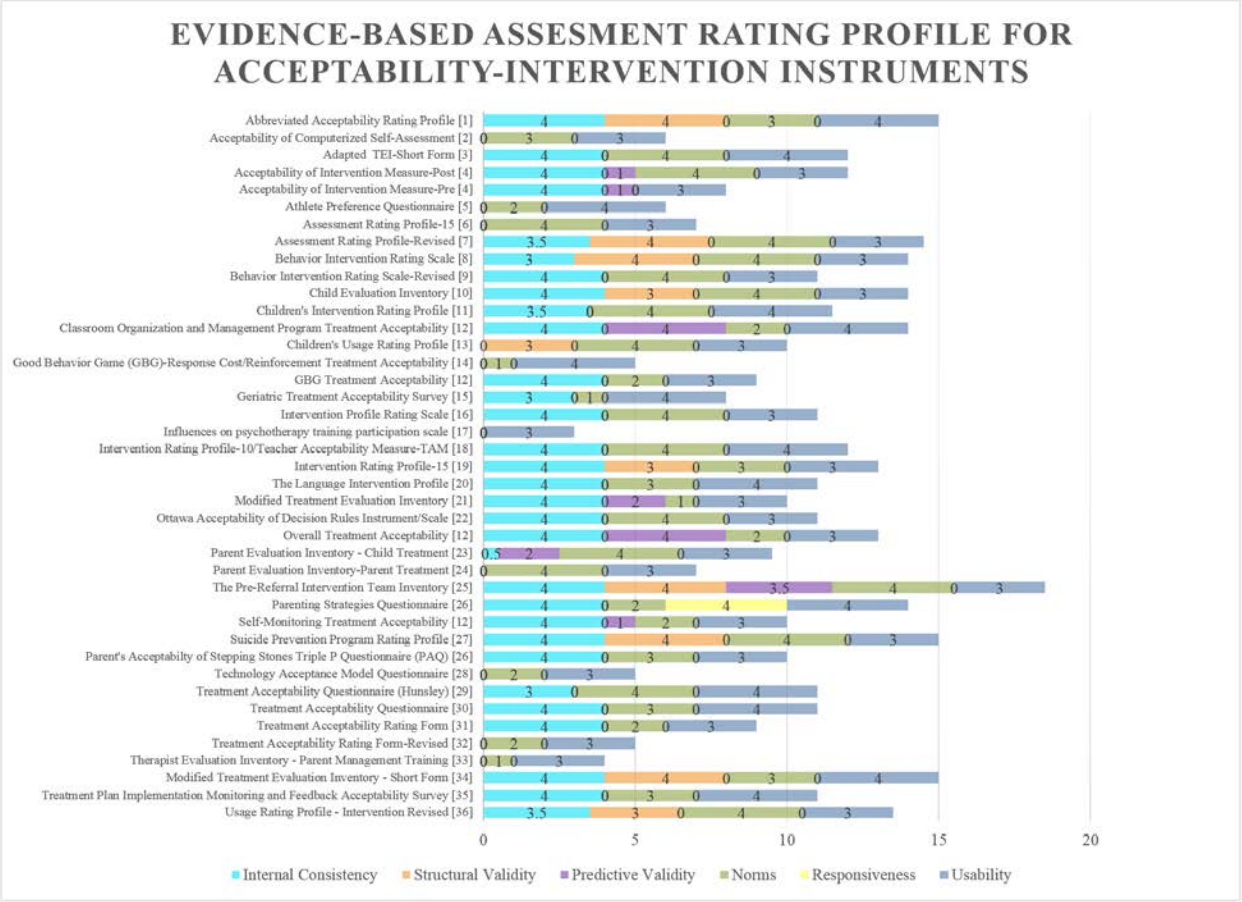

Figure 13: Acceptability-Implementation

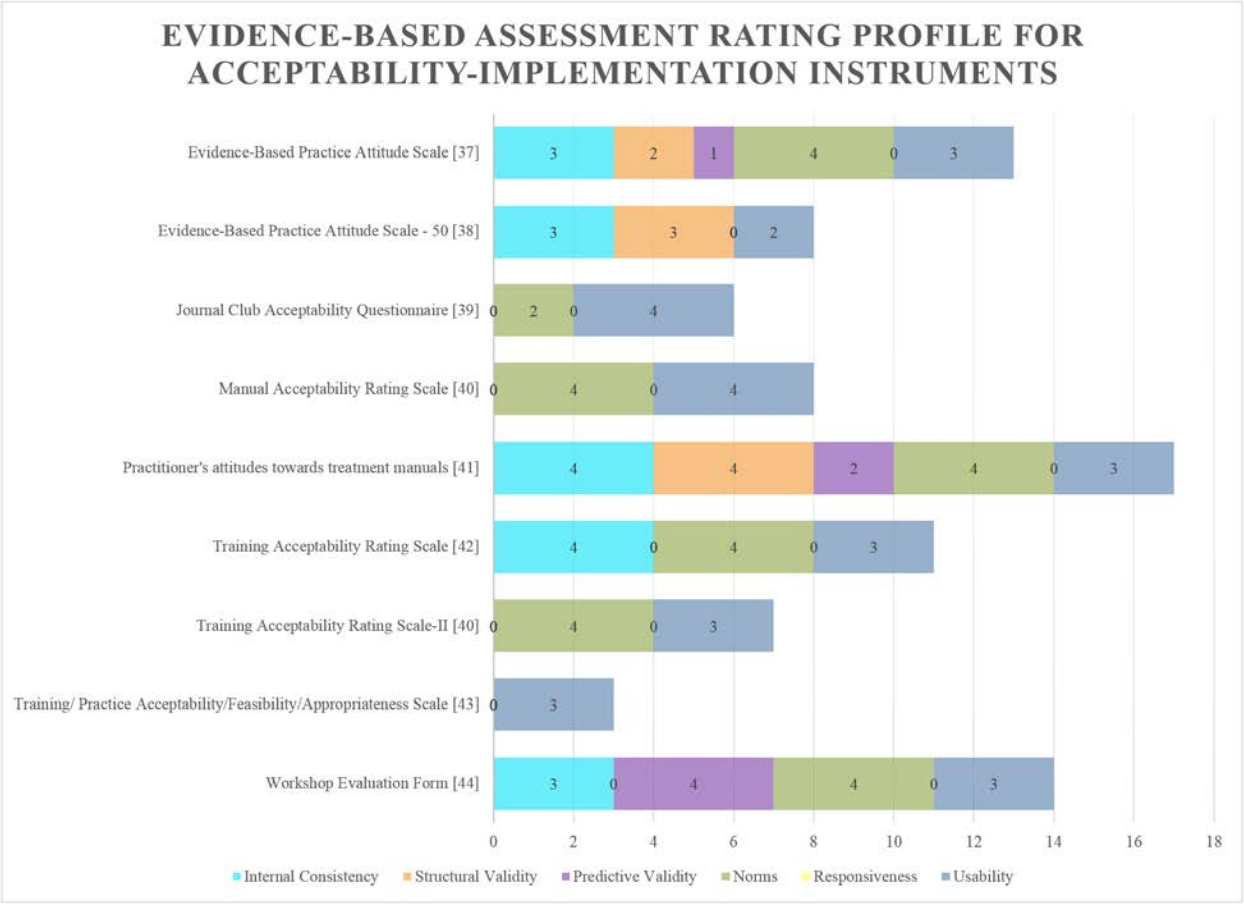

Figure 14: Adoption

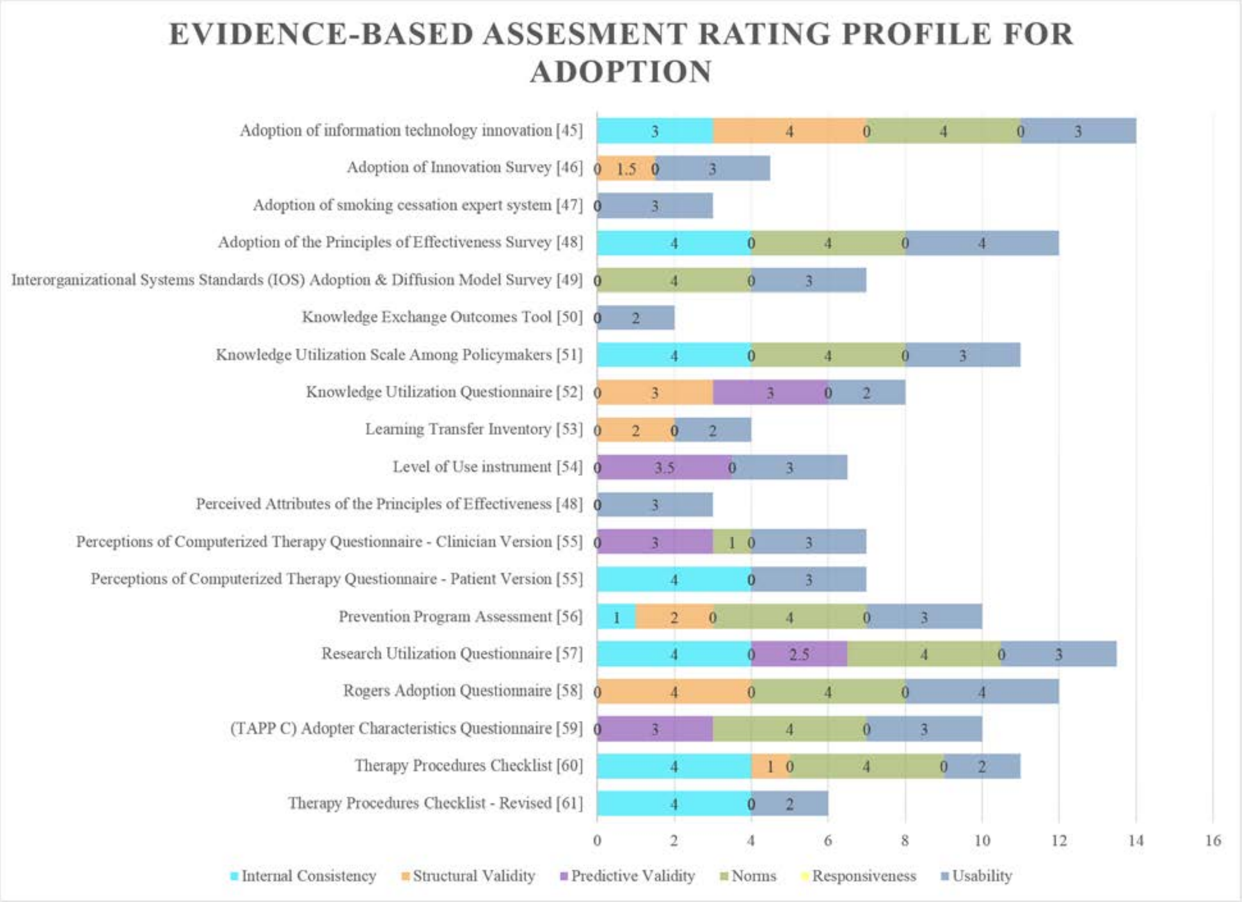

Figure 15: Appropriateness

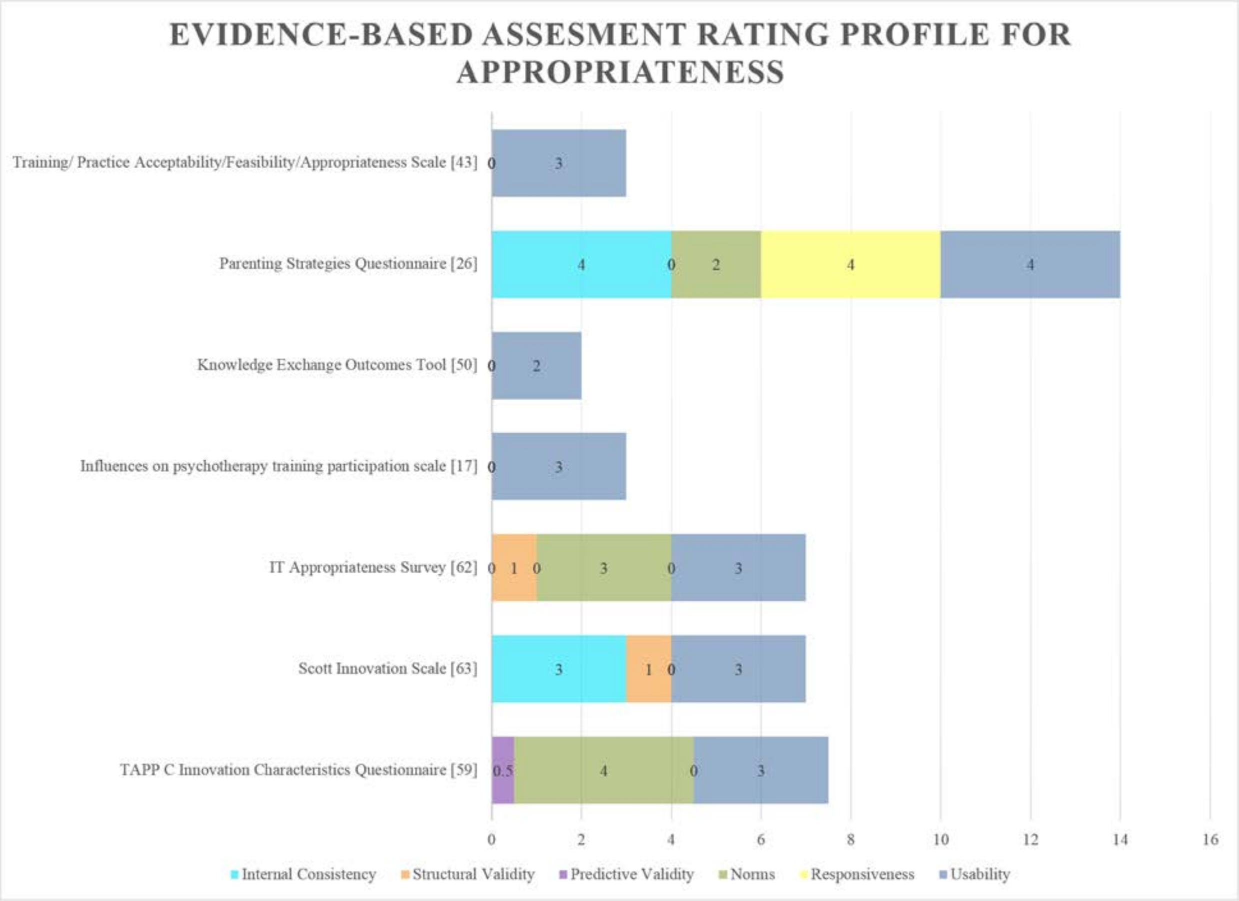

Figure 16: Cost

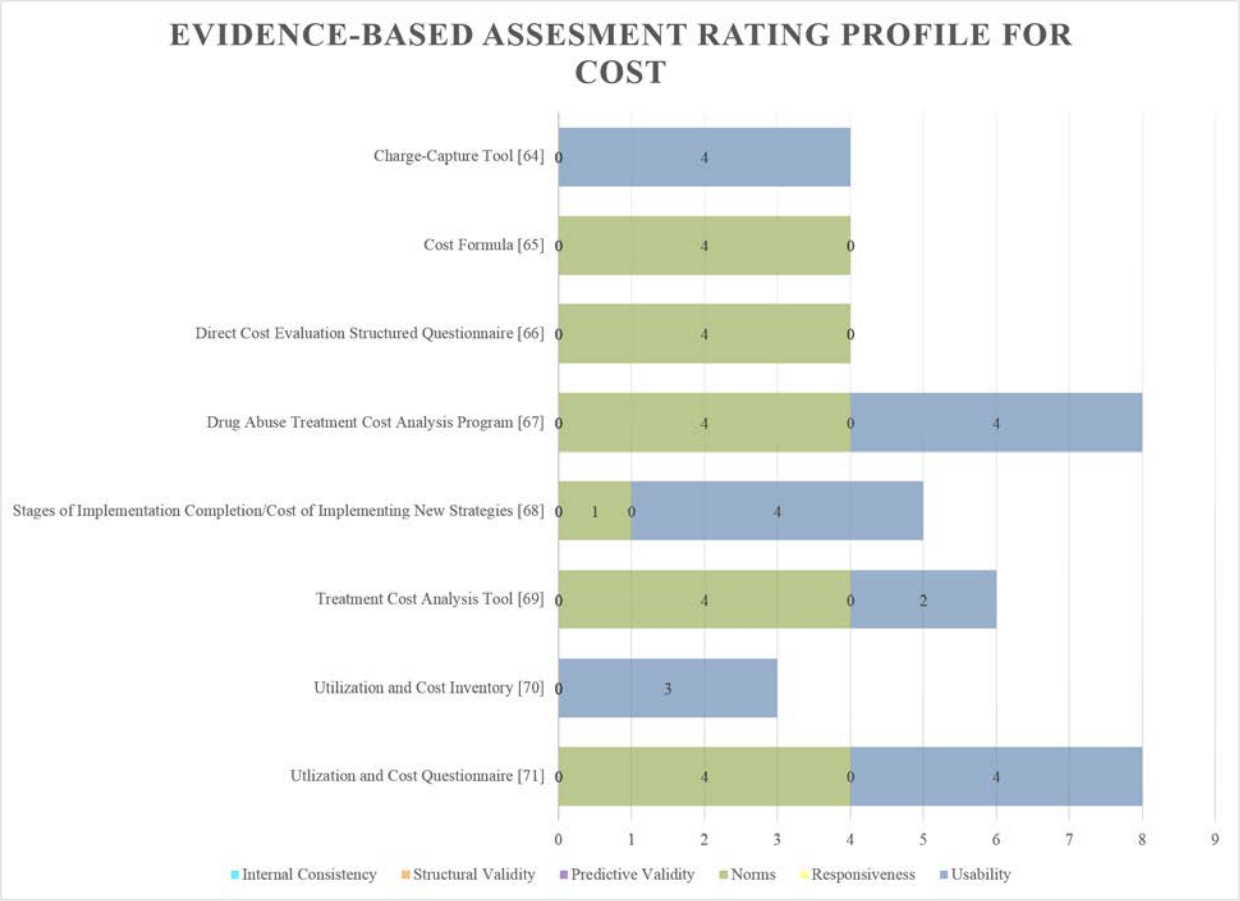

Figure 17: Feasibility

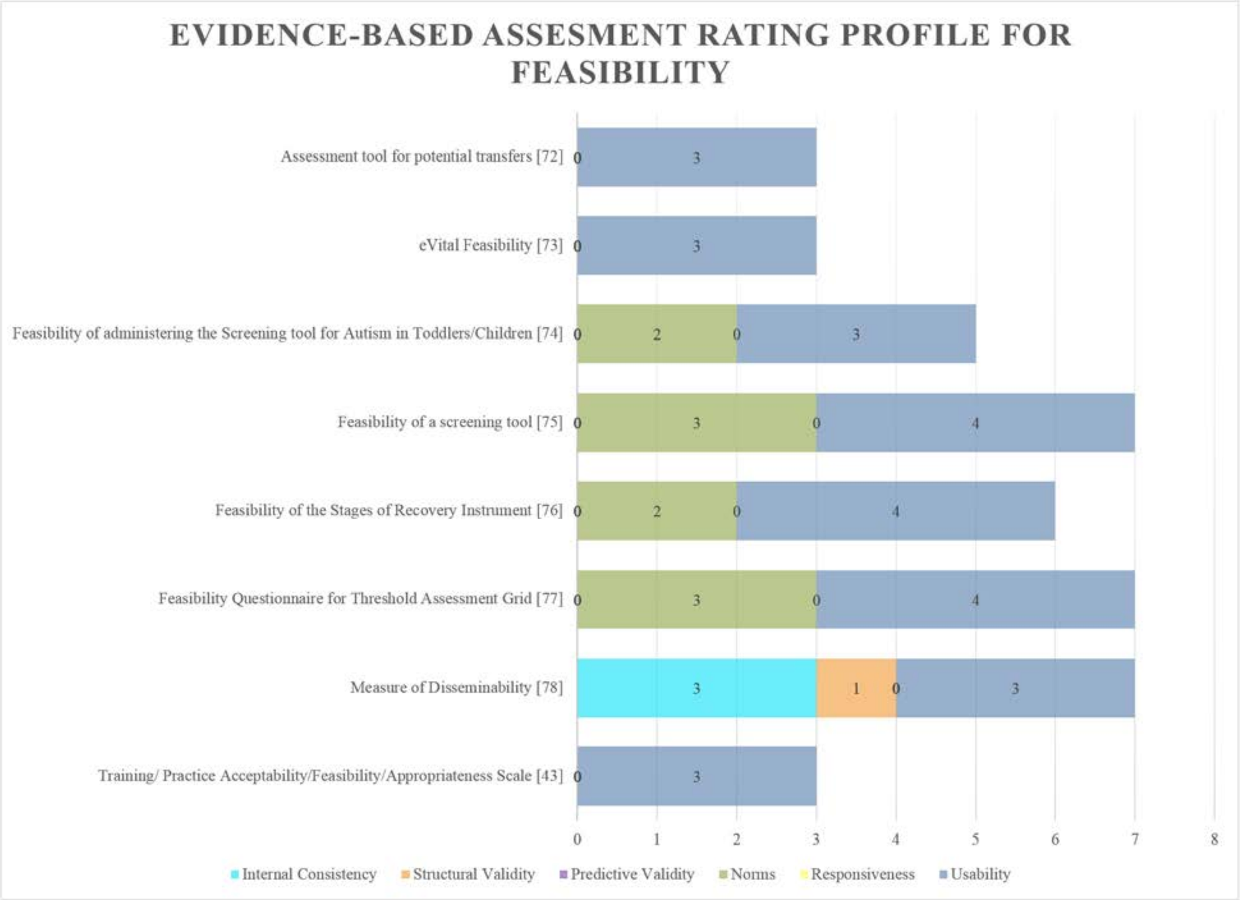

Figure 18: Penetration

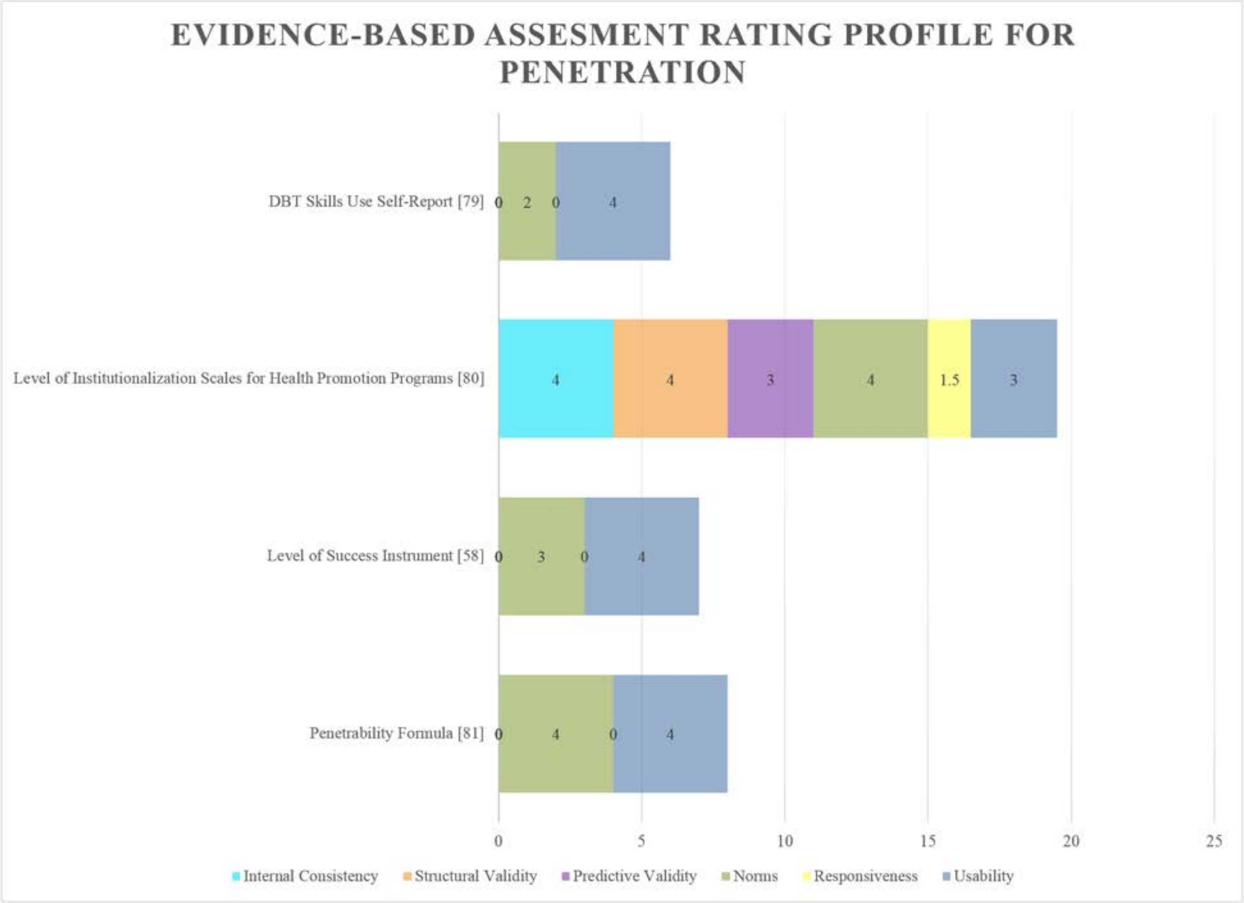

Figure 19: Sustainability

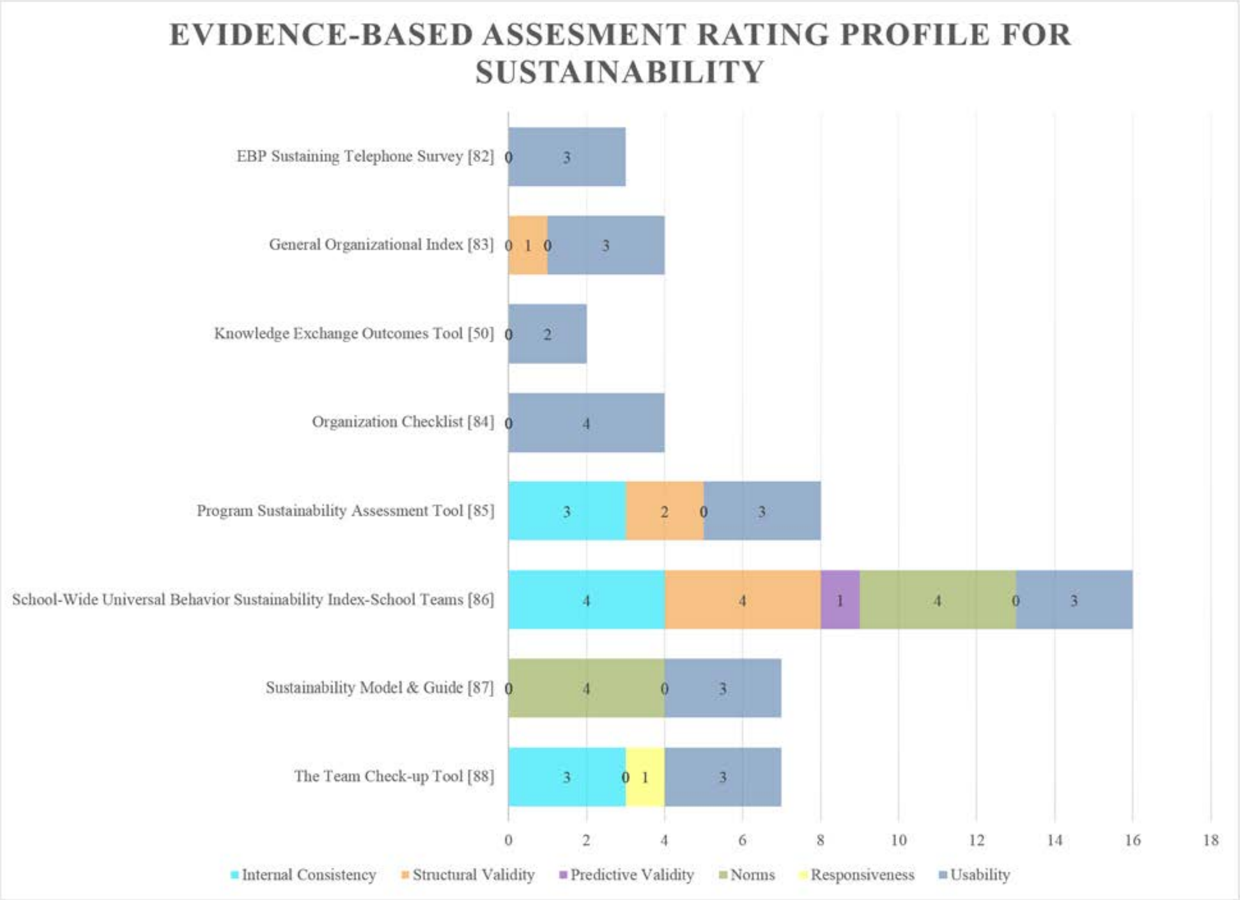

References

1. Tarnowski KJ, Simonian SJ: **Assessing treatment acceptance: The abbreviated acceptability rating profile**. *J Behav Ther Exp Psychiatry* 1992, **23**:101–106.

2. Bendtsen P, Timpka T: **Acceptability of computerized self-report of alcohol habits: a patient perspective**. *Alcohol Alcohol* 1999, **34**:575–580.

3. Johnston C, Hommersen P, Seipp C: **Acceptability of behavioral and pharmacological treatments for attention-deficit/hyperactivity disorder: Relations to child and parent characteristics**. *Behav Ther* 2008, **39**:22–32.

4. Henninger K: **Exploring the relationship between factors of implementation, treatment integrity and reading fluency**. 2010.

5. Maniar SD, Curry LA, Sommers-Flanagan J, Walsh JA, others: **Student-athlete preferences in seeking help when confronted with sport performance problems**. *Sport Psychol* 2001, **15**:205–223.

6. Connell Jr JE: **Constructing a math applications, curriculum-based assessment: An analysis of the relationship between applications problems, computation problems and criterion-referenced assessments**. *Ann Arbor MI ProQuest Inf Learn* 2006.

7. Eckert TL, Hintze JM, Shapiro ES: **Development and refinement of a measure for assessing the acceptability of assessment methods: The Assessment Rating Profile-Revised**. *Can J Sch Psychol* 1999, **15**:21–42.

8. Elliott SN, Treuting MVB: **The Behavior Intervention Rating Scale: Development and validation of a pretreatment acceptability and effectiveness measure**. *J Sch Psychol* 1991, **29**:43–51.

9. Semke CA: **Examining the efficacy of Conjoint Behavioral Consultation for health behaviors of children with obesity**. 2011.

10. Thurber S, Snow M, Thurber D: **Psychometric properties of the Child Evaluation Inventory.** *Psychol Assess J Consult Clin Psychol* 1990, **2**:206.

11. Elliott SN, Witt JC, Galvin GA, Moe GL: **Children's involvement in intervention selection: Acceptability of interventions for misbehaving peers.** *Prof Psychol Res Pract* 1986, **17**:235.

12. Tanol G: **Treatment Fidelity: Relation to Treatment Acceptability and Change Over Time**. *University of Minnesota*; 2010.

13. Briesch AM, Chafouleas SM: **Exploring Student Buy-In: Initial Development of an Instrument to Measure Likelihood of Children's Intervention Usage**. *J Educ Psychol Consult* 2009, **19**:321–336.

14. Tanol G, Johnson L, McComas J, Cote E: **Responding to rule violations or rule following: A comparison of two versions of the Good Behavior Game with kindergarten students**. *J Sch Psychol* 2010, **48**:337–355.

15. Lundervold D, Young L, Bourland G, Jackson T: **Psychometric properties of attitudinal measures of behavioral treatment in geriatric settings**. *Behav Interv* 1991, **6**:97–106.

16. Kutsick KA, Witt JC, Gutkin TB: **The impact of treatment development process, intervention type, and problem severity on treatment acceptability as judged by classroom teachers**. *Psychol Sch* 1991, **28**:325–331.

17. Lyon A: **Influences on Psychotherapy Training Participation Scale (IPTPS)**. 2010.

18. Power TJ, Hess LE, Bennett DS: **The acceptability of interventions for attention-deficit hyperactivity disorder among elementary and middle school teachers**. *J Dev Behav Pediatr* 1995, **16**:238–243.

19. Kratochwill TR, Elliott SN, Busse RT: **Behavior consultation: A five-year evaluation of consultant and client outcomes.** *Sch Psychol Q* 1995, **10**:87.

20. Turan Y: **Acceptability ratings of language interventions and reasoning as described by early childhood special education teachers**. *Early Child Dev Care* 2012, **182**:1371–1382.

21. Johnston C, Fine S: **Methods of evaluating methylphenidate in children with attention deficit hyperactivity disorder: acceptability, satisfaction, and compliance**. *J Pediatr Psychol* 1993, **18**:717–730.

22. Brehaut JC, Graham ID, Wood TJ, Taljaard M, Eagles D, Lott A, Clement C, Kelly A-M, Mason S, Kellermann A, others: **Measuring acceptability of clinical decision rules: validation of the Ottawa acceptability of decision rules instrument (OADRI) in four countries**. *Med Decis Making* 2009.

23. Kazdin AE, Bass D, Siegel T, Thomas C: **Cognitive-behavioral therapy and relationship therapy in the treatment of children referred for antisocial behavior.** *J Consult Clin Psychol* 1989, **57**:522.

24. Kazdin AE, Siegel TC, Bass D: **Cognitive problem-solving skills training and parent management training in the treatment of antisocial behavior in children.** *J Consult Clin Psychol* 1992, **60**:733.

25. Yetter G: **Assessing the Acceptability of Problem-Solving Procedures by School Teams: Preliminary Development of the Pre-Referral Intervention Team Inventory**. *J Educ Psychol Consult* 2010, **20**:139–168.

26. Whittingham K, Sofronoff K, Sheffield JK: **Stepping Stones Triple P: a pilot study to evaluate acceptability of the program by parents of a child diagnosed with an Autism Spectrum Disorder**. *Res Dev Disabil* 2006, **27**:364–380.

27. Eckert TL, Miller DN, DuPaul GJ, Riley-Tillman TC: **Adolescent suicide prevention: School psychologists' acceptability of school-based programs**. *Sch Psychol Rev* 2003, **32**:57–76.

28. Van Schaik P, Bettany-Saltikov JA, Warren JG: **Clinical acceptance of a low-cost portable system for postural assessment**. *Behav Inf Technol* 2002, **21**:47–57.

29. Hunsley J: **Development of the treatment acceptability questionnaire**. *J Psychopathol Behav Assess* 1992, **14**:55–64.

30. Krain AL, Kendall PC, Power TJ: **The role of treatment acceptability in the initiation of treatment for ADHD**. *J Atten Disord* 2005, **9**:425–434.

31. Reimers TM, Wacker DP: **Parents' ratings of the acceptability of behavioral treatment recommendations made in an outpatient clinic: A preliminary analysis of the influence of treatment effectiveness.** *Behav Disord* 1988.

32. Keleher KL: **Parental acceptance of behavioural treatments for children with autism**. 1999.

33. Myers SJ: *Relationship Between the Consultant-Parent Working Alliance and Ratings of the Consultation Process with Parents of Children Having Autism Spectrum Disorder*. ProQuest; 2008.

34. Kelley ML, Heffer RW, Gresham FM, Elliott SN: **Development of a modified treatment evaluation inventory**. *J Psychopathol Behav Assess* 1989, **11**:235–247.

35. Easton JE: **Teacher Acceptability of Treatment Plan Implementation Monitoring and Feedback Methods**. 2009.

36. Briesch AM, Chafouleas SM, Neugebauer SR, Riley-Tillman TC: **Assessing influences on intervention implementation: Revision of the usage rating profile-intervention**. *J Sch Psychol* 2013, **51**:81–96.

37. Aarons GA: **Mental health provider attitudes toward adoption of evidence-based practice: The Evidence-Based Practice Attitude Scale (EBPAS)**. *Ment Health Serv Res* 2004, **6**:61–74.

38. Aarons GA, Cafri G, Lugo L, Sawitzky A: **Expanding the domains of attitudes towards evidence-based practice: The Evidence Based Practice Attitude Scale-50**. *Adm Policy Ment Health Ment Health Serv Res* 2010, **39**:331–340.

39. Mazuryk M, Daeninck P, Neumann CM, Bruera E: **Daily journal club: an education tool in palliative care**. *Palliat Med* 2002, **16**:57–61.

40. Milne D: **Can we enhance the training of clinical supervisors? A national pilot study of an evidence-based approach**. *Clin Psychol Psychother* 2010, **17**:321–328.

41. Addis ME, Krasnow AD: **A national survey of practicing psychologists' attitudes toward psychotherapy treatment manuals.** *J Consult Clin Psychol* 2000, **68**:331.

42. Davis JR, Rawana EP, Capponi DR: **Acceptability of behavioral staff management techniques**. *Behav Interv* 1989, **4**:23–44.

43. Lyon A: **Training/Practice Acceptability/Feasibility/Appropriateness Scale**. 2011.

44. Bartholomew NG, Joe GW, Rowan-Szal GA, Simpson DD: **Counselor assessments of training and adoption barriers**. *J Subst Abuse Treat* 2007, **33**:193–199.

45. Moore GC, Benbasat I: **Development of an instrument to measure the perceptions of adopting an information technology innovation**. *Inf Syst Res* 1991, **2**:192–222.

46. Unsworth KL, Sawang S, Murray J, Sorbello T: **DEVELOPING AN INTEGRATIVE MODEL FOR UNDERSTANDING INNOVATION ADOPTION.** In *Academy of Management Proceedings*. *Volume 2009*. Academy of Management; 2009:1–6.

47. Hoving C, Mudde AN, de Vries H: **Intention to adopt a smoking cessation expert system within a self-selected sample of Dutch general practitioners**. *Eur J Cancer Prev* 2006, **15**:82–86.

48. Pankratz M, Hallfors D, Cho H: **Measuring perceptions of innovation adoption: the diffusion of a federal drug prevention policy**. *Health Educ Res* 2002, **17**:315–326.

49. Nelson ML, Shaw MJ: **The adoption and diffusion of interorganizational system standards and process innovations**. *Urbana* 2003, **51**:61801.

50. Skinner K: **Developing a tool to measure knowledge exchange outcomes**. *Can J Program Eval* 2007, **22**:49.

51. Landry R, Lamari M, Amara N: **The extent and determinants of the utilization of university research in government agencies**. *Public Adm Rev* 2003, **63**:192–205.

52. Chagnon F, Pouliot L, Malo C, Gervais M-J, Pigeon M-È: **Research article Comparison of determinants of research knowledge utilization by practitioners and administrators in the field of child and family social services**. *Implement Sci* 2010, **9**:19.

53. Chen K-N: **Library evaluation and organizational learning: A questionnaire study**. *J Librariansh Inf Sci* 2006, **38**:93–104.

54. McCormick LK, Steckler AB, McLeroy KR: **Diffusion of innovations in schools: a study of adoption and implementation of school-based tobacco prevention curricula**. *Am J Health Promot AJHP* 1995, **9**:210–219.

55. Carper MM, McHugh RK, Barlow DH: **The dissemination of computer-based psychological treatment: A preliminary analysis of patient and clinician perceptions**. *Adm Policy Ment Health Ment Health Serv Res* 2013, **40**:87–95.

56. Stamatakis KA, McQueen A, Filler C, Boland E, Dreisinger M, Brownson RC, Luke DA: **Measurement properties of a novel survey to assess stages of organizational readiness for evidence-based interventions in community chronic disease prevention settings**. *Implement Sci* 2012, **7**:65.

57. Champion VL, Leach A: **Variables related to research utilization in nursing: An empirical investigation**. *J Adv Nurs* 1989, **14**:705–710.

58. Steckler A, Goodman RM, McLeroy KR, Davis S, Koch G: **Measuring the diffusion of innovative health promotion programs**. *Am J Health Promot AJHP* 1992, **6**:214–224.

59. Henderson JL, MacKay S, Peterson-Badali M: **Closing the research-practice gap: Factors affecting adoption and implementation of a children's mental health program**. *J Clin Child Adolesc Psychol* 2006, **35**:2–12.

60. Weersing VR, Weisz JR, Donenberg GR: **Development of the therapy procedures checklist: A therapist-report measure of technique use in child and adolescent treatment**. *J Clin Child Adolesc Psychol* 2002, **31**:168–180.

61. Baumann BL, Kolko DJ, Collins K, Herschell AD: **Understanding practitioners' characteristics and perspectives prior to the dissemination of an evidence-based intervention**. *Child Abuse Negl* 2006, **30**:771–787.

62. Khazanchi D: **Information technology (IT) appropriateness: the contingency theory of "fit"and IT implementation in small and medium enterprises**. *J Comput Inf Syst* 2005, **45**:88–95.

63. Scott SD, Plotnikoff RC, Karunamuni N, Bize R, Rodgers W: **Factors influencing the adoption of an innovation: An examination of the uptake of the Canadian Heart Health Kit (HHK)**. *Implement Sci* 2008, **3**:41.

64. Nicoletti B: **Chart audit: is your practice billing what it should?**. *Fam Pract Manag* 2009, **16**:15–19.

65. Bloomquist ML, August GJ, Horowitz JL, Lee SS, Jensen C: **Moving from science to service: Transposing and sustaining the early risers prevention program in a community service system**. *J Prim Prev* 2008, **29**:307–321.

66. Allegri RF, Butman J, Arizaga RL, Machnicki G, Serrano C, Taragano FE, Sarasola D, Lon L: **Economic impact of dementia in developing countries: an evaluation of costs of Alzheimer-type dementia in Argentina**. *Int Psychogeriatr* 2007, **19**:705–718.

67. French MT, Bradley CJ, Calingaert B, Dennis ML, Karuntzos GT: **Cost analysis of training and employment services in methadone treatment**. *Evol Program Plan* 1994, **17**:107–120.

68. Saldana L, Chamberlain P, Bradford WD, Campbell M, Landsverk J: **The Cost of Implementing New Strategies (COINS): A method for mapping implementation resources using the Stages of Implementation Completion**. *Child Youth Serv Rev* 2014, **39**:177–182.

69. PAGES DE: **Treatment Cost Analysis Tool (TCAT) Data Entry and Review Guide**.

70. Kashner TM, Stensland MD, Lind L, Wicker A, Rush AJ, Golden RM, Henley SS: **Measuring use and cost of care for patients with mood disorders: the utilization and cost inventory**. *Med Care* 2009, **47**:184–190.

71. Kashner TM, Rush AJ, Altshuler KZ: **Measuring costs of guideline-driven mental health care: the Texas Medication Algorithm Project**. *J Ment Health Policy Econ* 1999, **2**:111–121.

72. King H, Tallman K, Huberman: **Assessment Tool for Potential Transfer**. 2004.

73. Alonso F, Walsh CO, Salvador-Carulla L, others: **Methodology for the development of a taxonomy and toolkit to evaluate health-related habits and lifestyle (eVITAL)**. *BMC Res Notes* 2010, **3**:83.

74. Kobak KA, Stone WL, Ousley OY, Swanson A: **Web-based training in early autism screening: Results from a pilot study**. *Telemed E-Health* 2011, **17**:640–644.

75. Hides L, Hides L, Lubman DI, Hides L, Lubman DI, Elkins K, Hides L, Lubman DI, Elkins K, Catania LS, others: **Feasibility and acceptability of a mental health screening tool and training programme in the youth alcohol and other drug (AOD) sector**. *Drug Alcohol Rev* 2007, **26**:509–515.

76. Weeks G, Slade M, Hayward M: **A UK validation of the Stages of Recovery Instrument**. *Int J Soc Psychiatry* 2010.

77. Slade M, Cahill S, Kelsey W, Powell R, Strathdee G, Valiakalayil A: **Threshold 3: the feasibility of the Threshold Assessment Grid (TAG) for routine assessment of the severity of mental health problems**. *Soc Psychiatry Psychiatr Epidemiol* 2001, **36**:516–521.

78. Trent LR: **Development of a Measure of Disseminability (MOD)**. *University of Mississippi*; 2010.

79. Dimeff LA, Woodcock EA, Harned MS, Beadnell B: **Can dialectical behavior therapy be learned in highly structured learning environments? Results from a randomized controlled dissemination trial**. *Behav Ther* 2011, **42**:263–275.

80. Goodman RM, McLeroy KR, Steckler AB, Hoyle RH: **Development of level of institutionalization scales for health promotion programs**. *Health Educ Behav* 1993, **20**:161–178.

81. Stiles PG, Boothroyd RA, Snyder K, Zong X: **Service penetration by persons with severe mental illness: How should it be measured?**. *J Behav Health Serv Res* 2002, **29**:198–207.

82. Aarons G: **EBP Sustaining Telephone Survey**. 2007.

83. Bond GR, Drake RE, Rapp CA, McHugo GJ, Xie H: **Individualization and quality improvement: two new scales to complement measurement of program fidelity**. *Adm Policy Ment Health* 2009, **36**:349–357.

84. Berliner L: **Organization Checklist**. .

85. Luke DA: **The Program Sustainability Assessment Tool: a new instrument for public health programs**. *Prev Chronic Dis* 2014, **11**.

86. McIntosh K, MacKay LD, Hume AE, Doolittle J, Vincent CG, Horner RH, Ervin RA: **Development and initial validation of a measure to assess factors related to sustainability of school-wide positive behavior support**. *J Posit Behav Interv* 2011:1098300710385348.

87. Maher L, Gustafson, D., Evans, A: **Sustainability and model guide**.

88. Chan KS, Hsu Y-J, Lubomski LH, Marsteller JA: **Validity and usefulness of members reports of implementation progress in a quality improvement initiative: findings from the Team Check-up Tool (TCT)**. *Implement Sci* 2011, **6**:115.
